# Curiosity-driven development of action and language in robots through self-exploration

**DOI:** 10.1126/sciadv.aee7533

**Published:** 2026-07-23

**Authors:** Theodore J. Tinker, Kenji Doya, Jun Tani

**Affiliations:** Okinawa Institute of Science and Technology, Okinawa, Japan.

## Abstract

Infants acquire language with generalization from minimal experience, whereas large language models require billions of training tokens. What underlies efficient development in humans? We investigated this problem through experiments wherein robotic agents learn to perform actions associated with imperative sentences (e.g., push red cube) via curiosity-driven self-exploration. Our approach amortizes active inference using Q-learning, enabling intrinsically motivated developmental learning. The simulations reveal key findings corresponding to observations in developmental psychology. (i) Generalization improves markedly as the scale of compositional elements increases. (ii) Curiosity-driven exploration enables faster learning. (iii) Rote pairing of sentences and actions precedes compositional generalization. (iv) Exception handling induces U-shaped developmental performance, a pattern like representational redescription in child language learning. These results suggest that curiosity-driven active inference accounts for how intrinsically motivated sensorimotor-linguistic learning supports scalable compositional generalization and exception handling in humans and artificial agents.

## Teaser

Robots learn to perform actions associated with sentences via curiosity-driven self-exploration.

## INTRODUCTION

A central question in both cognitive science and artificial intelligence is how humans and artificial systems can acquire competencies for language and action developmentally despite having access to only limited learning experiences. This question is exemplified in human infants, who achieve substantial generalization with sparse input. This is a stark contrast to large-scale models that rely on massive training corpora to reach similar capabilities. This raises the issue of what mechanisms enable such efficient developmental learning.

### Compositionality, generalization, and exception handling

From the perspective of developmental psychology, infants acquire language through rich interaction with their embodied environments. Tomasello’s “verb-island” hypothesis (*1*) argues that children initially learn verbs in specific, isolated contexts before generalizing across broader linguistic structures with compositionality. He also emphasized the importance of embodiment in language acquisition, suggesting that grounding linguistic symbols in sensorimotor experiences is fundamental to language learning (*1*). This view aligns with other studies in developmental psychology highlighting the role of compositionality and generalization in language acquisition (*2*–*4*).

In linguistic theory, compositionality refers to the principle that the meaning of a complex expression is determined by the meanings of its constituent parts together with the rules used to combine them (*5*–*7*). This principle implies that linguistic representations are structured and rule governed, typically exhibiting hierarchical organization. A key cognitive consequence of compositional structure is systematicity, namely the capacity to understand and produce novel but structurally related expressions by recombining familiar elements (*8*). In this view, systematic generalization across combinations of verbs, adjectives, and nouns reflects the presence of an underlying compositional organization, rather than mere associative pairing. Thus, generalization is not itself the definition of compositionality but a behavioral manifestation of compositional representations, enabling learners to flexibly construct and interpret utterances that have not been directly encountered (*9*).

In the present study, compositionality is operationalized in terms of such systematic generalization across novel combinations of known elements (*8*, *9*). We emphasize that this operationalization captures only one aspect of compositionality and does not aim to model the full hierarchical and formal semantic structure of natural language, as defined in classical linguistic theory (*5*, *6*). Rather, our focus is on the developmental emergence of systematic recombination capacity in embodied agents as a minimal and tractable step toward understanding the origins of compositionality. A nontrivial problem is that although the number of possible compositions grows multiplicatively with the vocabulary size (i.e., number of verbs × number of adjectives × number of nouns), children achieve generalization after experiencing only a small subset of learning examples. This suggests that the effective sample complexity could be proportional to the sum of elements rather than their product. This phenomenon is closely related to the “poverty of the stimulus” problem articulated by Chomsky (*10*), which asks how learners generalize so effectively given severely sparse input.

Beyond these, it is well known that children can develop the capacity for exception handling, a hallmark of flexible cognition. In human development, exceptions such as irregular verbs or inconsistent mappings often produce nonmonotonic, U-shaped learning trajectories: Children first apply a correct form, then overgeneralize it (producing errors), and lastly recover the correct rule. This pattern has been widely interpreted as evidence of internal representational reorganization or representational redescription (*11*). Computationally, such U-shaped performance has been demonstrated in models of language acquisition and rule learning (*12*–*16*). Developmentally, these phenomena reflect the tension between rote memorization, generalization, and the later refinement of exception rules.

### Synthetic robotics study using active inference

How can humans develop the capacity for compositionality as systematic generalization even with exception handling through learning from sparse input? To investigate this question, one promising approach is to reconstruct developmental learning processes in machines and robots. The field of developmental robotics has long pursued this line of research, aiming to replicate human-like learning trajectories in embodied systems (*17*–*20*). However, relatively few studies have focused on the development of language and motor control under conditions of stimulus poverty. Existing work has primarily examined associative mappings between linguistic input and motor commands in one-shot or supervised batch learning schemes (*21*–*24*). These approaches neglect the self-directed, developmental context of infant learning.

In this study, we propose a novel scheme for self-exploratory learning of robots by integrating active inference (*25*–*27*) and reinforcement learning (*28*, *29*). Our integration of active inference and reinforcement learning can be regarded as amortizing inference, sometimes known as learning to infer or deep active inference (*30*–*32*). However, we have gone further than usual amortization schemes by replacing extrinsic reward alone with expected free energy, which includes expected information gain. This means that agents are effectively rewarded for being curious or, more simply, for learning to be curious. Our approach to integrate reinforcement learning with active inference was originally inspired by the work of Kawahara *et al.* (*33*). In our model, originally introduced in (*34*), motor commands are reinforced by two intrinsic rewards: curiosity (seeking unpredictable sensory consequences) and motor entropy (seeking random movements). Motor commands are also reinforced by extrinsic rewards for successfully achieving goals specified by given imperative sentences. Our previous experiments in maze navigation demonstrated that the combination of curiosity and motor entropy is crucial for enhancing self-exploration, as agents achieved substantially improved exploratory behaviors under this dual-intrinsic reward scheme. Our approach aligns with broader research on self-exploration in machine learning, in which agents are intrinsically rewarded for taking motor actions whose consequences are unpredictable and therefore lead to the greatest information gain, i.e., have an epistemic value (*35*, *36*).

**Fig. 1.**
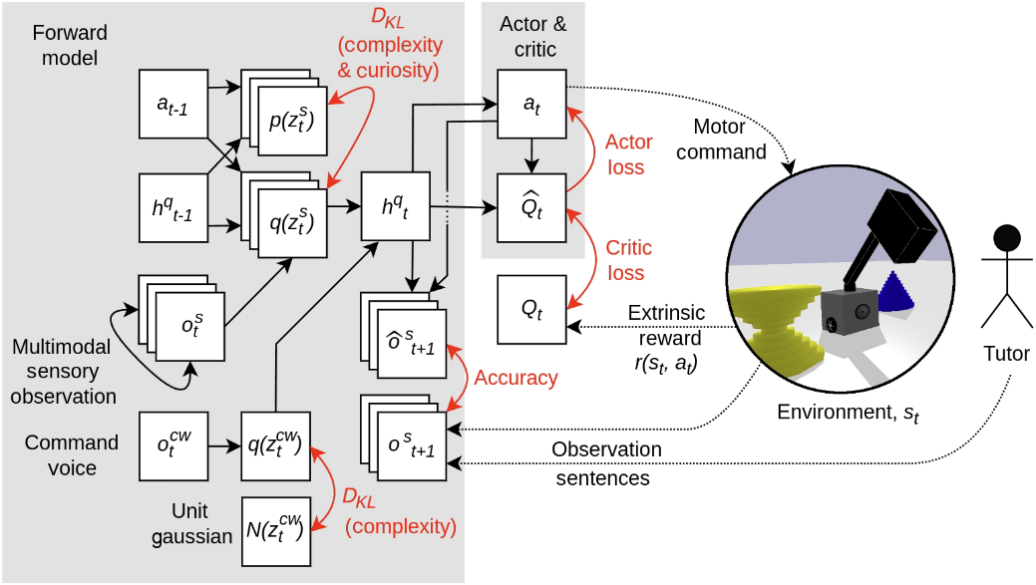
Proposed model architecture. The model consists of a forward model, actor, and critic. Variables include the following: world state $s_{t}$, sensory observation $o_{t}^{s}$, command voice $o_{t}^{\mathrm{cw}}$, motor command $a_{t}$, *Q* value $Q_{t}$, latent control variables $h_{t}^{q}$, random latent variables $z_{t}$, prior for sensations $p\left(z_{t}^{s}\right)$, approximated posterior for sensations $q\left(z_{t}^{s}\right)$, and approximated posterior for command voice $q\left(z_{t}^{\mathrm{cw}}\right)$.

A simulated mobile robot equipped with a manipulator arm, vision sensor, and distributed tactile sensors learns to generate motor movements in response to imperative sentences (command voice) presented during each trial. These sentences are systematically composed of verbs, adjectives, and nouns, enabling evaluation of generalization performance under different levels of compositional complexity. The model architecture used in this study is based on our previous work (*34*) with key modifications to accommodate multimodal sensorimotor integration. (See details in Materials and Methods.) Figure 1 presents the model architecture, which is composed of three main components: a forward model, an actor part, and a critic part. The forward model learns to predict the next sensation $o_{t+1}^{s}$ on the basis of the current sensation $o_{t}^{s}$, command voice $o_{t}^{\mathrm{cw}}$, and the executed motor command $a_{t}$. The sensation includes pixel-based vision, tactile sensation, and arm joint proprioception. To address the hidden state problem and probabilistic nature of the environment, the prediction is performed contextually and stochastically using the random latent variables $z_{t}^{s}$ and latent control variables $h_{t}^{q}$, wherein the former with Gaussian probability distribution is inferred by minimizing the variational free energy, while the latter is just computed forwardly using sampling of the inferred random latent variable. Here, $p\left(z_{t}^{s}\right)$ is the prior probabilistic distribution of the random latent variables before observing the current sensation, while $q\left(z_{t}^{s}\right)$ is the posterior one after observation of the current sensation caused by the current motor command execution, as will be detailed later. The latent control variables were shared for each sensory modality, while the random latent variables were allocated separately. This separation of random latent variables was necessary for the system to deal with different types of sensory modalities simultaneously. The actor module generates the next motor command $a_{t}$ on the basis of the latent control variable $h_{t}^{q}$, which integrates current sensation $o_{t}^{s}$. On the basis of $a_{t}$ and $h_{t}^{q}$, the critic generates $\hat{Q}$, which is a prediction of the *Q* value defined with Eq. 4. While the critic learns to make accurate predictions about future rewards in *Q*, the actor learns to produce motor commands, which maximize the critic’s predictions $\hat{Q}$. (In the actual architecture, there is another sensory input, so-called tutor-feedback voice. This additional input will be detailed later.)

The overall flow is as follows: With a command sentence given by the tutor, the robot attempts to achieve the specified goal by generating a sequence of motor commands. Meanwhile, the forward model predicts the next sensation by inferring the posterior probability distribution $q\left(z_{t}^{s}|o_{t},h_{t-1}\right)$ of the random latent variable $z^{s}$. This inference is conducted by minimizing the variational free energy *F* (Eq. 1). This consists of the complexity term represented by Kullback-Leibler divergence (KLD) between the approximated posterior and the prior and the accuracy term under the free energy principle. (See more details on the variational free energy and expected free energy in the “Free energy principle, active inference, and Kawahara model” section of the Supplementary Materials.)

$$F_{\psi ,t}=\underset{\text{Complexity}}{\underbrace{D_{\mathrm{KL}}\left[q\left(z_{t}|o_{t},h_{t-1}\right)\|p\left(z_{t}|h_{t-1}\right)\right]}}-\underset{\text{Accuracy}}{\underbrace{E_{q\left(z_{t}\right)}\left[\text{log}p\left(o_{t+1}|h_{t}\right)\right]}}$$ (1)

The forward model is trained iteratively by optimizing its learning parameters ψ in the direction of minimizing the evidence free energy. The actor learns to generate motor command sequences in the direction of minimizing the expected free energy *G* (Eq. 2) through reinforcement learning. This consists of the complexity term, extrinsic reward term, and motor entropy term

$$G\left(a_{t}\right)=-\underset{\text{Curiosity}}{\underbrace{D_{\mathrm{KL}}\left[q\left(z_{t}|o_{t},h_{t-1}\right)\|p\left(z_{t}|h_{t-1}\right)\right]}}-\underset{\text{Extrinsic reward}}{\underbrace{r\left(s_{t},a_{t}\right)}}-\underset{\text{Entropy}}{\underbrace{H\left[\pi_{\phi }\left(a_{t}|h_{t}\right)\right]}}$$ (2)

Minimizing evidence free energy *F* minimizes the complexity term, while minimizing expected free energy *G* maximizes the same complexity term. This means that motor commands are generated in the direction of maximizing the information gain attained after execution of the motor command, which is represented by KLD between the approximated posterior and the prior. This generates curiosity-driven exploration wherein the agent seeks out previously unencountered sensorimotor experiences. On the other hand, the learning parameters in the forward model are updated in the direction of minimizing the same complexity term, representing the latent conflict that is generated by novel experiences encountered during curiosity-driven exploration. Therefore, both processes of self-exploration and the forward model learning are complementing each other as if in competition. In other words, inference about latent states causing observations is trying to minimize complexity, while learning which actions to take is trying to maximize complexity expected after acting.

Note also that the expected information gain is evaluated using observations at each time point. This can be compared with the usual expressions for expected free energy in which an expectation over future observations is used. In general, observations in the future are random variables, and the sign of the KLD subtending complexity switches so that expected information gain is maximized. However, because we are amortizing or learning to minimize expected free energy, we can evaluate the expected complexity on the basis of current observations, thereby enabling the agent to learn to be information-seeking. In other words, expected free energy is not used to select the next action; it is used as an objective function to learn what action to take next.

### Limitations

Although the present study draws inspiration from developmental psychology, the resemblance between our model and human infant language development remains limited. Human infants undergo extensive sensorimotor and social learning for at least the first 6 months of life before exhibiting substantial linguistic competence (*37*, *38*). Early language acquisition typically progresses from single-word utterances to two-word combinations and only later to more complex multiword constructions (*1*, *3*). Around the onset of combinatorial speech, infants often exhibit a vocabulary spurt, acquiring new words at a substantially rapid pace (*39*). These developmental milestones occur within a richly scaffolded social environment, including joint attention, imitation, and caregiver feedback, which substantially reduce the complexity of the learning problem (*40*–*42*). In contrast, our model operates in a simplified environment without prior nonlinguistic developmental stages, social scaffolding, or progressive linguistic complexity.

Furthermore, the present framework assumes learning from scratch without incorporating innate cognitive biases, architectural constraints, or evolutionary priors that are widely argued to support human language and action acquisition (*10*, *43*, *44*). Human learners likely benefit from species-specific perceptual, social, and representational predispositions shaped by evolutionary history. Our model does not attempt to capture such innate structures. Instead, it investigates how compositionality as systematic generalization can emerge from curiosity-driven sensorimotor learning under minimal assumptions. Therefore, while the present results demonstrate a mechanistic account of systematic recombination in embodied agents, they should not be interpreted as a comprehensive model of human language development. Rather, they provide a simplified testbed for examining how associative learning of language and action based on active inference may contribute to the emergence of structured generalization.

### Hypothesis

This study therefore tested the following hypotheses through simulation experiments: H1: Generalization performance improves markedly as the scale of compositionality in the task increases. H2: Curiosity augmented with motor entropy enhances the performance of developmental learning. H3: In the early phase, actions are generated only for exactly learned imperative sentences, but in later phases, the system generalizes to novel, unlearned compositions. H4: Primitive actions are acquired earlier, followed by more complex, prerequisite-dependent actions. H5: Exception handling rules can be acquired through exploratory learning, exhibiting U-shaped developmental performance similar to that observed in human development.

## RESULTS

### Task description

**Fig. 2.**
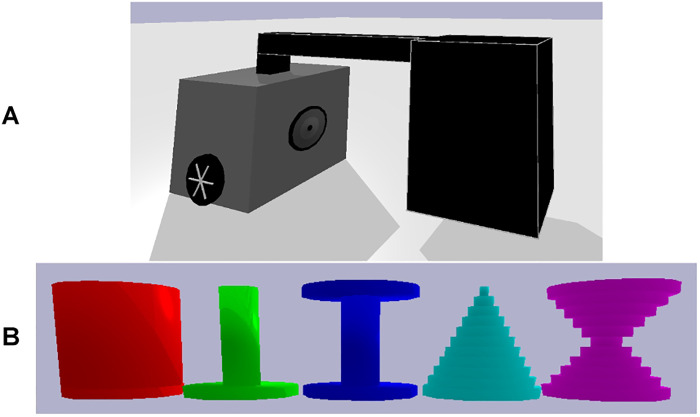
Simulated robot and a set of objects to act on. (**A**) The robot has two wheels and an arm with two joints. The design is similar to a truck crane. (**B**) Left to right: a red pillar, a green pole, a blue dumbbell, a cyan cone, and a magenta hourglass. The color yellow is not pictured here.

We created a robot like a truck crane in a physics simulator along with a set of objects with five different shapes, each of which can take six different colors (see Fig. 2). The robot can maneuver by controlling the velocity of left and right wheels independently and also can move its arm by controlling the rotation velocity of the yaw and pitch joint angles for acting on the objects. A camera with 16 by 16 pixels was fixed to the body for visual sensation. Sixteen touch sensors were distributed in the body and the arm, and rotation angles for the yaw and pitch were sensed as proprioception.

For each trial episode, a task goal was given in terms of an imperative sentence composed of a verb, adjective, and noun. Possible words used for them are shown in Table 1. In the beginning of each episode, two objects were located at random positions in the arena, wherein one object was the one specified in the imperative sentence and the other was the one with randomly selected color and shape combination among possible ones.

**Table 1.** English words. The English words used for imperative sentences specifying goals.

Verb	Adjective	Noun
Watch	Red	Pillar
Be near	Green	Pole
Touch the top	Blue	Dumbbell
Push forward	Cyan	Cone
Push left	Magenta	Hourglass
Push right	Yellow	

At each step, the robot receives visual sensation, proprioception for the arm, tactile sensation, and two types of voices: the command voice and the tutor-feedback voice. The command voice takes the format of the imperative sentence described previously and is delivered continuously at every step from the beginning. On the other hand, the feedback voice arrives whenever the robot achieves one of possible goals even if the achieved goal is not the imperative sentence told by the command voice, and it informs which goal has been actually achieved in the same format with the command voice. This potentially enhances the forward model’s learning about its own action as associated with linguistic representation. Last, when the goal specified by the command voice is achieved, a reward is provided. Each trial episode ran for 30 steps or was terminated when the specified goal is achieved. Then, the robot is trained with a random batch of 32 episodes.

### Effects of curiosity: Experiment 1

This experiment examined the effects of different levels of curiosity to the developmental learning processes using the basic setup. In the basic setup, full compositions of words (Table 1) were used to generate the imperative sentences. However, the training was conducted using only 60 imperative sentences (33%) of 180 possible sentences. One hundred twenty untrained sentences were used for generalization testing. For 10 robots with different random seeds, the complete developmental learning process was iterated for 60,000 epochs. Generalization testing with unlearned imperative sentences was conducted every 50 epochs.

The experiment was conducted by changing the levels of curiosity. Given that the random latent variables are computed separately for each sensory modality, the complexity or curiosity can be computed for each sensory modality. Three levels of curiosity were considered in computing expected free energy *G*: no curiosity, wherein none of the curiosity terms for sensory modalities are included; sensory-motor curiosity, wherein the curiosity terms only for vision, tactile sensation, and proprioception are included; and all curiosity, wherein the curiosity terms for all sensory modalities including feedback voice are included.

**Fig. 3.**
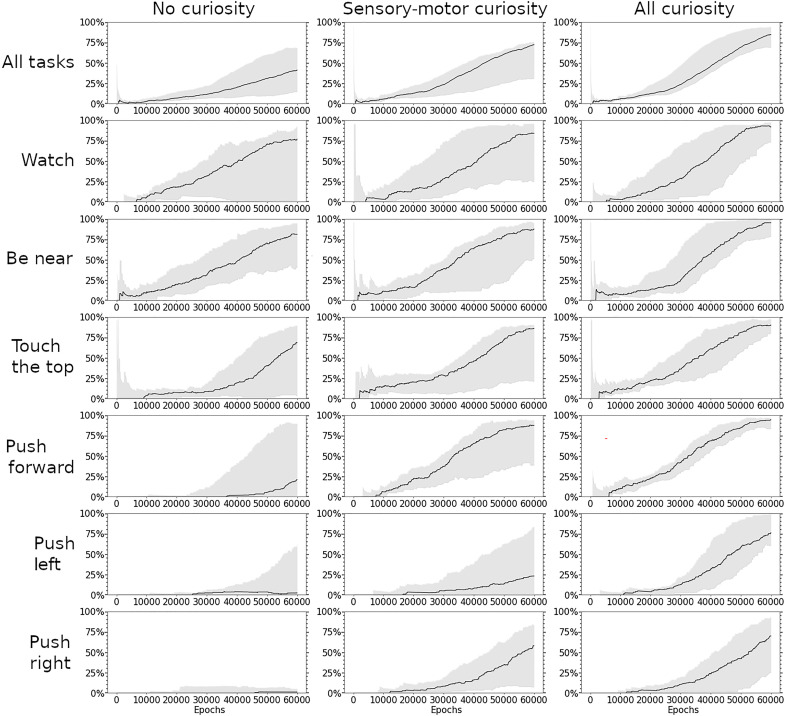
Rolling success rates for unlearned goals. Comparing agents with different levels of curiosity. Shaded areas represent 99% confidence intervals for the 10 agents.

Figure 3 shows the development of the generalization testing performances in terms of success rate for goals specified by unlearned imperative sentences, which are plotted for different action categories with different levels of curiosity. The plots show that the performance was improved significantly as the curiosity level was increased. The case of all actions with the all-curiosity level shows that the average success rate for unlearned goals reached a quite high value of 85.1%, even though the learning was conducted only for 33% of all possible compositions.

It can also be observed in Fig. 3 that some action categories develop more slowly than others in the all-curiosity condition. In particular, “push right” and “push left” exhibit slower development compared to the other action categories. This may be attributed to the relatively higher behavioral complexity associated with these actions. In contrast, no clear or significant differences in development speed are observed among the remaining actions, namely “watch,” “be near,” “push forward,” and “touch the top.”

**Fig. 4.**
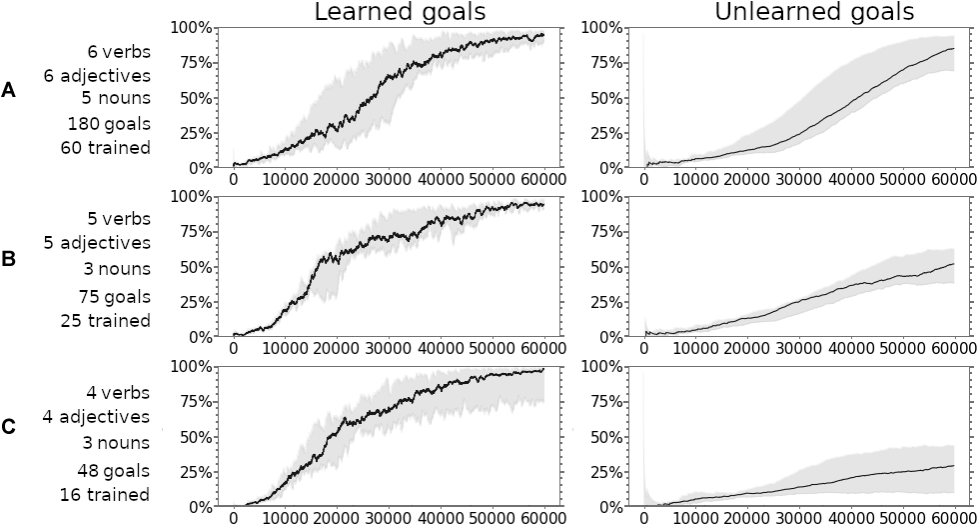
Rolling success rates for learned and unlearned goals with different compositionality scales. (**A**) Agents trained with all six verbs, all six adjectives, and all five nouns. (**B**) Agents trained with five verbs, five adjectives, and three nouns. (**C**) Agents trained with four verbs, four adjectives, and three nouns. Shaded areas represent 99% confidence intervals for the 10 agents.

Next, Fig. 4A shows the success rate comparison between learned and unlearned goals under the all-curiosity condition for each action category. These plots show that the test performance for learned goals developed substantially faster than the case for the unlearned goals. This indicates that actions are generated only for exactly learned compositional imperative sentences in the early phase, but the system generalizes to novel, unlearned ones in the later phase.

It is noted that the performance of the no-curiosity case is not completely saturated at 60,000 epochs (41%). Therefore, we conducted another simulation of this case up to 120,000 epochs to examine whether its performance could further improve. It appears that the performance of the all-curiosity case at 60,000 epochs (85.1%) is comparable to that of the no-curiosity case at 120,000 epochs (80.8%; see fig. S5). This result indicates that the no-curiosity case can gain a generalization performance similar to the all-curiosity case, but its developmental speed is much slower than that of the all-curiosity case.

For a comparison with the proposed model, we implemented a conventional baseline architecture on the basis of the Soft Actor-Critic algorithm (*45*), in which both the actor and critic networks are equipped with GRU (gated recurrent unit) recurrent layers (*46*). Details of this baseline model are provided in the “Experiment 1” section in the Supplementary Materials. Although figs. S6 and S7 show the best results achieved through various trials with different hyperparameter settings, its performance was substantially lower than that of the proposed model using active inference for curiosity-driven exploration.

Movie S1 shows examples of the behaviors of robots with the all-curiosity level. It can be seen that in the intermediate phase of development, the robot often acts with play-like behavior without achieving specified goals. In the final phase of development, it quickly and accurately achieves its goals.

### Further analysis

**Fig. 5.**
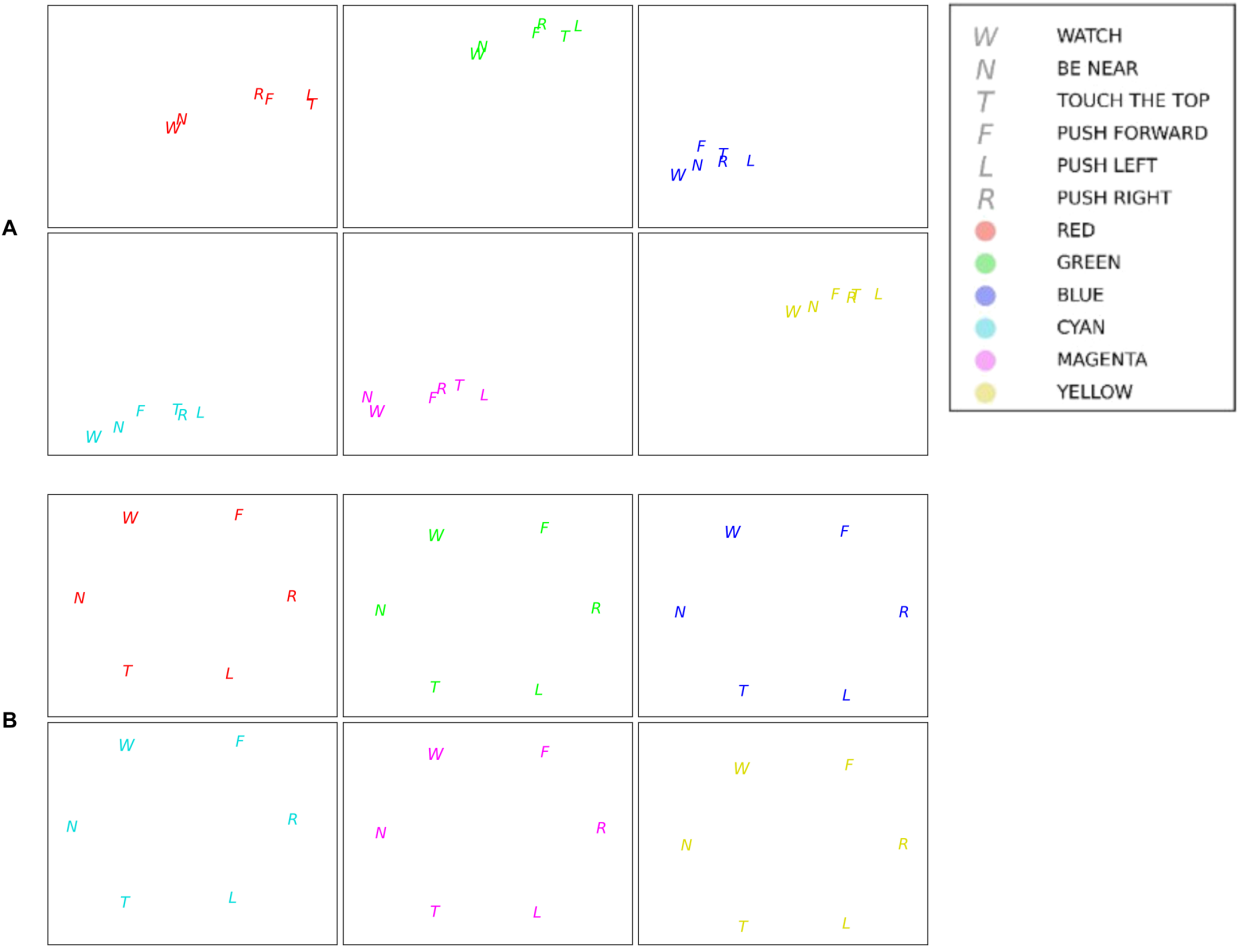
PCA for language latent variables in the case of all curiosity. PCA of latent variables corresponding to the command voice. (**A**) Halfway through development (after 30,000 epochs). (**B**) Complete development (after 60,000 epochs).

Post hoc analyses were conducted to characterize the internal representations developed. Figure 5 (A and B) shows the analysis of the internal representation by applying principal components analysis (PCA) to the approximated posterior latent states corresponding to the command voice input at 30,000 and 60,000 epochs in the development, respectively. Each panel shows the alignment of the principal component values among different verbs with the same adjective (color) averaged over all different object nouns.

In the case of full development (after 60,000 epochs) shown in Fig. 5B, it can be seen that the alignment of verbs repeats for all different colors. This means that the internal representation for command sentences is factorized with different dimensions of verb and adjective/color after the full development. On the other hand, in the case of halfway through development (after 30,000 epochs) shown in Fig. 5A, the alignments of verbs across different colors are partially broken, especially among “touch the top” (T), “push left” (L), and “push right” (R). From these observations, it can be said that the structure for systematicity compositionality can be developed gradually in the course of developmental learning.

**Fig. 6.**
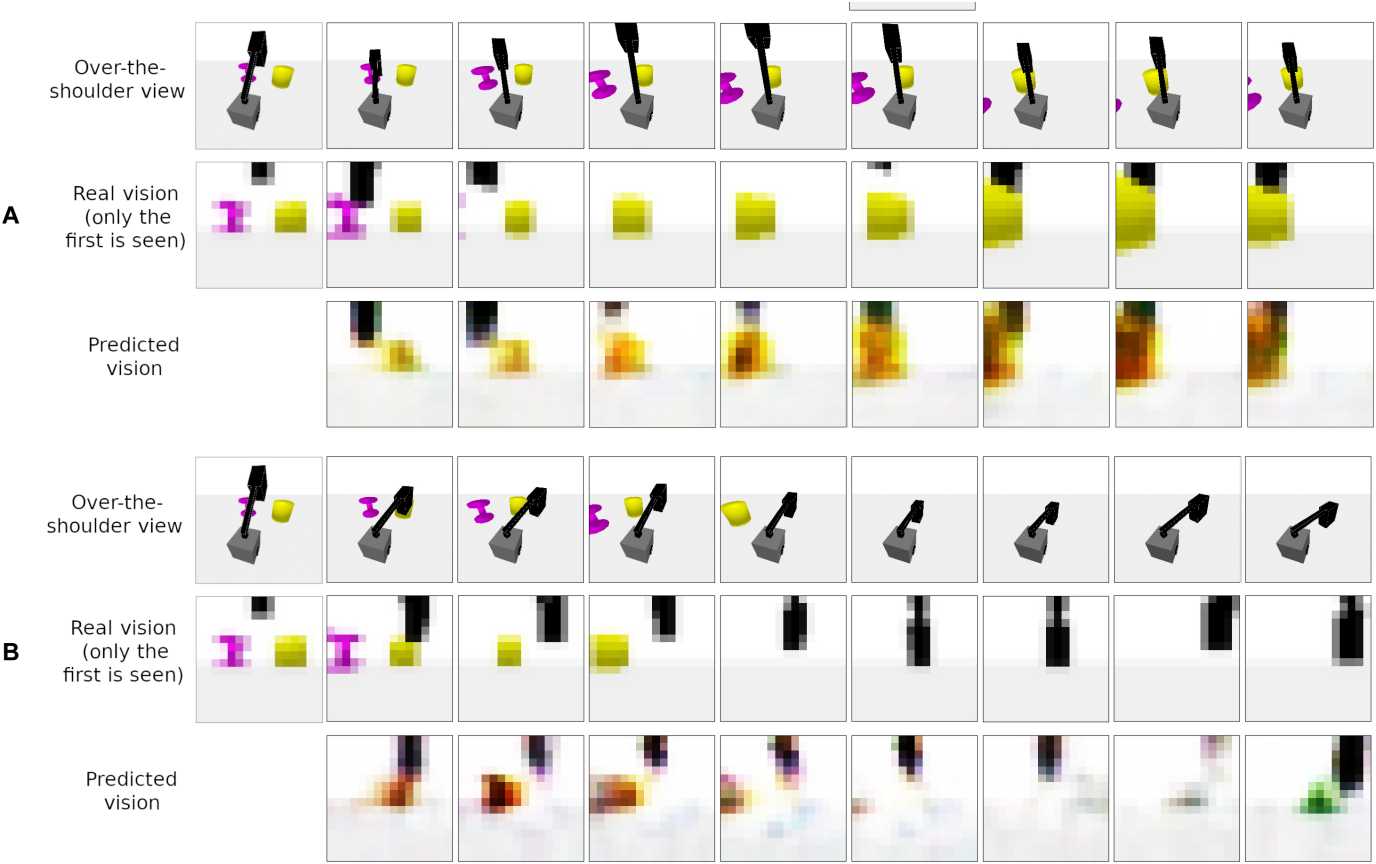
Mental plans generated by the robot. This robot is commanded to touch the top of the yellow pillar. The first row displays the ground truth from a view over the robot’s right shoulder. The second row displays the visual sequence of the ground truth. The third row shows the visual sequence of mental planning. (**A**) Case for the end of complete developmental learning and (**B**) for the case of halfway through development.

In the current model, the robot’s knowledge of the environment should become richer over the course of exploratory learning. For confirmation of this idea, we examined the capability of the robots in generating mental plans for achieving goals without accessing the sensory inputs, except the initial step for an episode trial as compared between the half-trained case and the fully trained case. A robot’s mental planning can be visualized by allowing it to receive real sensory observation only at the initial step, after which the robot must rely entirely on its own internal predictions. In this setting, the robot views its predicted sensory observations as if they are true inputs. This process may be likened to a dreamlike state or hallucinatory simulation, in which the robot mentally simulates future events on the basis of its internal model of the world. Figure 6A illustrates a simulation example for the fully trained case. The robot was commanded to touch the top of the yellow pillar. In that figure, the first row shows the ground truth environment from a view behind the robot’s shoulder. The second row shows what the robot would truly observe if it were not in this planning setting. The third row shows the robot’s look ahead predictions for visual observations, which it interprets as if they are real. It can be seen that even with only the first step sensory observation, the robot could generate mostly accurate future look-ahead prediction for sensation as well as motor command. These predictions are sufficiently accurate for the robot to maintain an internal conceptualization of the environment and complete its command in the case of the end of the developmental learning. Figure 6B illustrates the same robot after only half of its training in the same scenario. In this case, the robot’s predictions are inaccurate, causing it to wander and view an object that does not actually exist.

### Effects of scale in compositions: Experiment 2

This experiment examines the effects of scales of compositionality in learned examples to the generalization performance. For this purpose, experiments were conducted using a reduced number of words for generating imperative sentences. While the previous basic setup used sentences composed of six verbs, six adjectives, and five object nouns as the full scale case, the middle scale case was prepared with five verbs, five adjectives, and three object nouns, and the small scale case was prepared with four verbs, four adjectives, and three object nouns. The exact words used for each setup are listed in Table 2. For all scaling cases, only one-third was used for learning examples, while the remaining two-thirds were used for generalization testing. Other experimental conditions were the same as in Experiment 1.

**Table 2.** Words for training in three ways. Verbs, adjectives, and nouns that are used for training agents in three different ways.

Name	Verbs	Adjectives	Nouns
Largest vocabulary	Watch	Red	Pillar
	Be near	Green	Pole
	Touch the top	Blue	Dumbbell
	Push forward	Cyan	Cone
	Push left	Magenta	Hourglass
	Push right	Yellow	
Reduced vocabulary	Watch	Red	Pillar
	Be near	Green	Pole
	Push forward	Blue	Dumbbell
	Push left	Cyan	
	Push right	Magenta	
Smallest vocabulary	Watch	Red	Pillar
	Push forward	Green	Pole
	Push left	Blue	Dumbbell
	Push right	Cyan	

The experimental results are shown in Fig. 4. It can be seen that although the learned goal test cases show equally high performance for all scales of compositionality, generalization testing for the unlearned goal case shows that the success rate in the final trial decreases significantly (85.1 to 29%) as the compositionality scale decreases. This indicates that the generalization performance severely depends on the scale of compositionality in learning examples.

### Exception rule handling: Experiment 3

This experiment examined how robots can acquire exception handling rules through developmental learning. While most command-action mappings were preserved, the commands “watch magenta pillar” and “be near green pole” were swapped: Success required performing the other goal, not the one commanded. These mismatches required the robot to override its learned generalized knowledge.

This simulation experiment was conducted using the same model parameters used in the previous experiments with 10 robots with 60,000 epochs of developmental trials. At the end of development, the average success rate among 10 robots was 83% for the learned goals, 74% for unlearned goals, and 51% for the exception handling cases. The average success rate for the exception handling cases is not so high (only some individuals are successful), which should be reasonable by considering the task complexity.

**Fig. 7.**
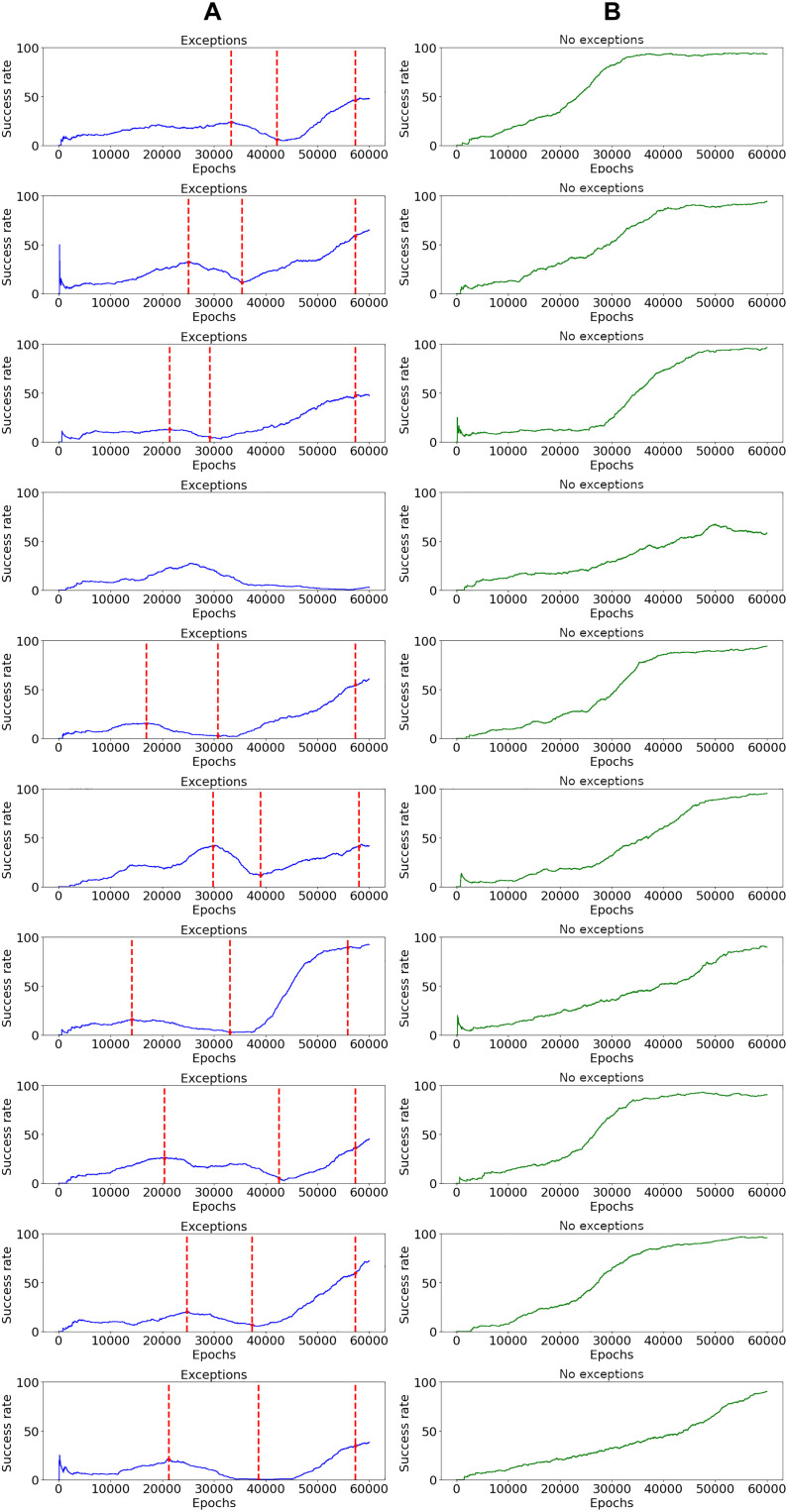
Comparison of the performance curve with and without applying the exception handling rules for 10 individuals. (**A**) Development of success rate in achieving two goals that are swapped as exceptions. Red vertical lines depict the peaks and valleys of the learning, as defined in Supplementary Text. (**B**) Development of success rate in achieving the same two goals without applying the exception handling rules.

Figure 7A shows the rolling success rate for achieving the exception goals for each of 10 individual robots, while Fig. 7B shows the success rate for the same goals but without applying the exception handling rules. The final success rate for the exception handling case is diverse, ranging from 5 to 90%, as can be seen in Fig. 7A. It was also found that 9 of 10 robots trained with these exceptions exhibit characteristic U-shaped curves: early success, followed by a drop, and eventual recovery with a higher success rate than the earlier one. In contrast, a monotonic increase in success rate can be seen in all 10 individuals in the case of learning without the exception handling rules. Statistical comparisons (detailed in Supplementary Text) confirm that U-shaped patterns are significantly more prevalent in the exception condition than in the control (P=0.0001).

**Fig. 8.**
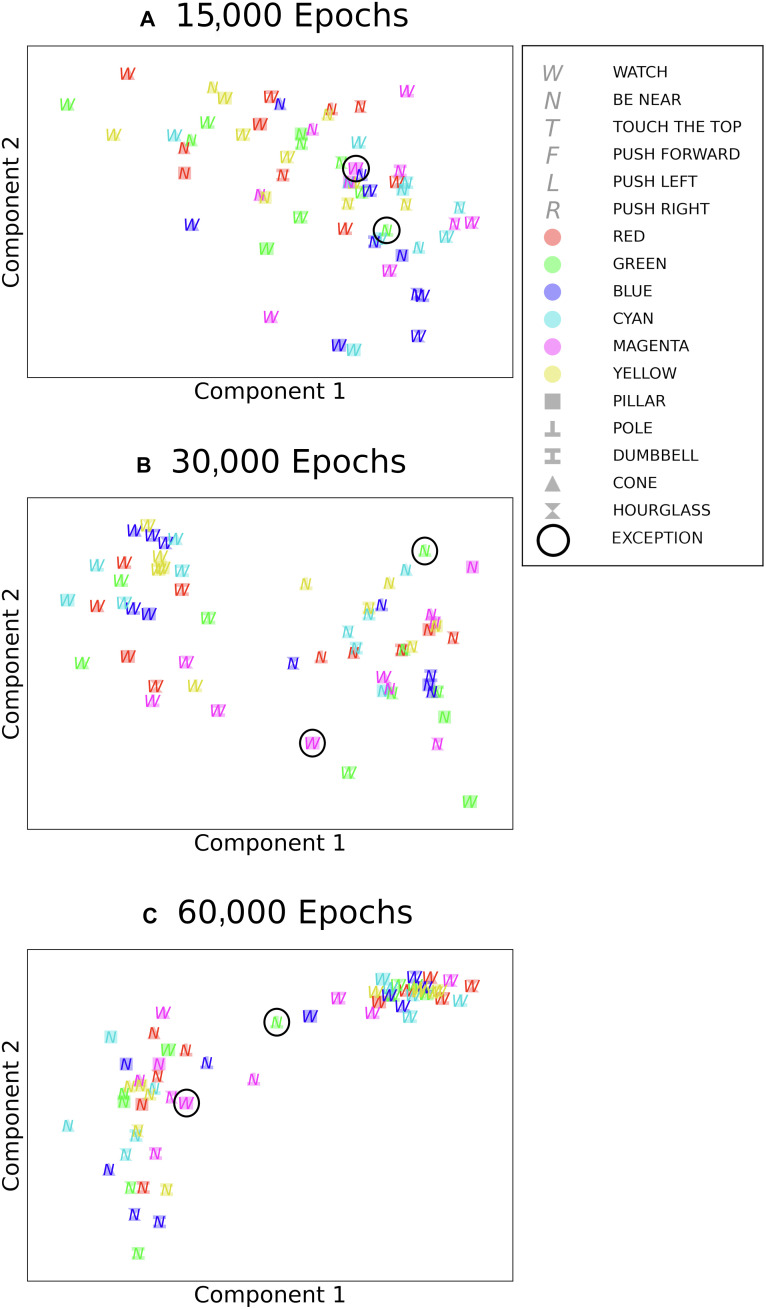
PCA for language latent variables for an individual developed with the exception handling rules. (**A**) Plot at 15,000 epochs, (**B**) plot at 30,000 epochs, and (**C**) plot at 60,000 epochs, wherein the circled “N” and circled “W” denote the sentences applied with exception handling.

In Fig. 8, PCA illustrates how the internal representations of goals with the tasks “watch” or “be near” in the seventh robot in Fig. 7A evolve in a manner consistent with the U-shaped success rates. (Note that goals with other tasks are not shown for better visualization purpose.) In Fig. 8A, early in training, goal embeddings are muddled without a clear structure, reflecting a learning phase with minimal generalization. In Fig. 8B, midway in training, the exception command “watch magenta pillar” is embedded with other “watch” goals, while “be near green pole” clusters are embedded with other “be near” goals despite these associations being incorrect. These observations indicate that overgeneralization has occurred. In Fig. 8C, late in training, “watch magenta pillar” is now embedded near “be near” goals and “be near green pole” near “watch” goals, indicating that the robot has correctly handled these exceptions as swapped pairs. Here, it can be said that the representational redescription took place in the course of developmental learning of exception handling rules.

## DISCUSSION

This study investigated how robots can develop action and language through self-exploration by amortizing active inference with deep learning. The experiments were designed to test five specific hypotheses, and the results provide clear support for each.

### H1: Generalization is enhanced by compositional scale

The experiments confirmed that larger vocabularies of verbs, adjectives, and nouns led to greater generalization. Robots trained with richer compositional repertoires achieved higher success rates on unlearned actions, whereas smaller vocabularies constrained generalization severely. Our previous study (*24*) on supervised training of language and action for an arm robot also showed that compositional generalization improved as the size of verb-noun combinations in training increased. However, that work was limited in scale, examining only cases from 3 × 3 to 5 × 8 verb-noun combinations, where success rates for unlearned goals improved only from 57 to 71% under the condition of 80% training. By contrast, the present study examined much broader scaling, ranging from 48 to 180 possible compositions, while using only 33% of them for training. Under these conditions, generalization performance improved markedly from near 25 to 85%. This contrast highlights the fact that scale plays a critical role in enhancing generalization and that curiosity-driven developmental learning provides a more powerful mechanism than supervised schemes under conditions of limited input. The finding also connects to the classical “poverty of the stimulus” problem raised by Chomsky (*10*), as it shows how compositionality enables powerful generalization from sparse training data. Once, we hypothesized that necessary training size could be proportional to summation of the number of words that appeared for each dimension instead of multiplication of it for all dimensions if compositionality size increases (*24*). This hypothesis becomes more plausible given the results in this current study, which should be confirmed in much more scaled experiments in the future.

### H2: Curiosity combined with motor entropy enhances developmental learning

These results support H2, indicating that curiosity-driven exploration facilitates the development of structured internal representations that enable compositional generalization under conditions of sparse experience. In particular, agents equipped with intrinsic motivation acquire such representations more efficiently and with fewer training epochs compared to those without curiosity. However, additional experiments extending the training of the no-curiosity condition to 120,000 epochs revealed that comparable levels of compositional generalization can eventually be achieved given sufficiently prolonged experience. This suggests that while curiosity is not strictly necessary for the emergence of structured representations, it plays a critical role in accelerating learning and improving sample efficiency. Therefore, the primary contribution of curiosity-driven exploration in the present framework lies in facilitating faster and more efficient acquisition of compositional structure rather than being the sole mechanism enabling it.

### H3: Generalization follows rote learning

The results again align with this hypothesis: In early phases, the robot succeeds only on exactly learned sentence-action pairs, and it is only over time that the robot begins to generalize to novel combinations of known elements. This mirrors developmental patterns in infants, who often begin with rigid pairings before achieving broader generalization (*1*). Tomasello’s “verb-island” hypothesis (*1*), for example, emphasizes that children initially acquire verbs in isolated contexts before generalizing across broader structures, in the same way that this robot acquired trained goals first before generalizing to untrained goals. Gerken and Knight (*47*) demonstrated that 10- to 11-month-old infants can generalize from just four linguistic examples under favorable conditions. Moreover, Gerken *et al.* (*48*) provide evidence that infants may generalize even from a single unexpected example, suggesting that hypothesis-driven generalization can follow minimal exposure. These studies lend developmental credence to our observed progression from rote mapping toward flexible compositional generalization.

### H4: Primitive actions precede complex actions

Regarding the fourth hypothesis, “primitive actions precede complex actions,” our results provide only partial support. As shown in Fig. 3, in the all-curiosity condition, some relatively complex actions, such as “push left” and “push right,” emerged more slowly than other behaviors, which is broadly consistent with the hypothesis. However, among the remaining actions, namely “watch,” “be near,” “push forward,” and “touch the top,” no clear or statistically significant differences were observed in their developmental speeds despite differences in their presumed levels of action complexity. This pattern differs somewhat from the original expectation of a monotonic relationship between action complexity and acquisition speed. One possible interpretation is that factors other than structural complexity, such as environmental affordance, reward structure, or perceptual accessibility, may play an important role in shaping the developmental trajectory of actions.

### H5: Exception handling rules exhibit U-shaped development

The results from Experiment 3 provide strong support for this hypothesis. When robots were trained with two swapped command-action mappings, U-shaped performance trajectories were observed more frequently than robots trained without these exceptions. Our analysis of the latent variables in the model network showed that overgeneralization takes place in the middle of development and such an internal representation is redescribed to accommodate the exception handling rules later. This nonmonotonic, U-shaped performance trajectory mirrors a well-established phenomenon in developmental psychology, in which children first succeed on irregular forms, later overgeneralize newly learned rules (e.g., producing “goed” for “went”), and ultimately reorganize their internal representations to master both rules and exceptions. Classic accounts interpret these dynamics as evidence for representational redescription, a restructuring of internal knowledge that enables more abstract, generative representations (*11*).

Computational modeling has long shown that such U-shaped learning can emerge naturally from error-driven or distributed representations, including Rumelhart and McClelland’s connectionist model of English past-tense acquisition (*12*), the multilayer perceptron models of Plunkett and Marchman (*13*, *14*), and broader frameworks in computational developmental psychology (*16*). Our robot simulations demonstrate that an analogous process arises in curiosity-driven active inference: The agent first relies on rote pairings, then applies generalized compositional mappings that overwrite earlier exceptions, and lastly reconstructs its latent representation to encode the exception rules correctly.

The parallels with infant development, including rote-to-generalization progression, prerequisite learning, the role of vocabulary scale, and representation redescription, suggest that the mechanisms implemented here capture some essential aspects of developmental psychology. More broadly, these results strengthen the view that reconstructing developmental processes in robots can offer insights into the “poverty of the stimulus” problem, showing how powerful generalization can arise from limited input when guided by intrinsic motivation, structured experience, and the principles of predictive coding and active inference (*17*, *22*, *24*, *49*).

While the present model demonstrates the emergence of compositional generalization under curiosity-driven self-exploration, several limitations should be noted. First, although a comparison with a Soft Actor-Critic baseline suggests that the proposed architecture benefits from the inclusion of a forward model for structuring latent representations, the current study is not intended to establish performance superiority over alternative artificial intelligence models but rather to elucidate qualitative developmental characteristics under varying conditions such as compositional scale and intrinsic motivation. In addition, the learning process is relatively slow, requiring on the order of 60,000 trials to achieve stable performance, indicating limited efficiency under the present setting.

Second, the present framework provides only a simplified abstraction of human development. Unlike human infants, whose language acquisition is preceded by rich sensorimotor and social experience and supported by caregiver scaffolding and developmental staging, our model isolates a minimal setting to investigate how compositional generalization and exception handling can emerge from intrinsically motivated exploration. Accordingly, the model should be understood as a controlled platform for examining core mechanisms rather than a comprehensive account of human language development.

These limitations naturally point to several important directions for future research. One key extension is to move beyond the current one-directional communication paradigm between tutors and robots. In the present study, learning is guided solely by externally provided commands and feedback, whereas human development is characterized by interactive and bidirectional communication (*50*, *51*). Future work should therefore incorporate mechanisms by which robots can actively solicit guidance, request clarification, or negotiate task difficulty, enabling more adaptive and socially grounded learning through interaction with caregivers.

Another important direction concerns improving learning efficiency by introducing structured developmental progression. The current model assumes learning from scratch without prior knowledge, whereas human learners acquire perceptual categories and basic motor repertoires before engaging in more complex compositional tasks. Incorporating pretrained perceptual and motor primitives, together with curriculum learning in which simpler tasks are acquired before more complex combinations (*1*, *52*), may substantially accelerate learning and improve generalization. More broadly, integrating both staged learning and social interaction will be essential for bridging the gap between the present minimal framework and the richness of human developmental processes.

Another promising future direction concerns the development of robot-robot communication through the evolution of language. Previous research has explored this possibility from different perspectives: Steels (*53*) introduced the framework of “language games” to study the emergence of shared vocabularies among agents, Li and Miikkulainen (*54*) investigated the evolution of artificial language through evolutionary reinforcement learning, and Taniguchi *et al.* (*55*) proposed the emergence of symbols using a collective predictive coding approach. While these studies have demonstrated the possibility of emergent communication in an impressive manner, they still remain limited in that they only considered the emergence of object labeling or naming, whereas the evolution of action-related language, such as verbs, has been much less explored. In this context, the current study based on active inference could be extended to address the evolution of dynamic linguistic structures, including verbs. Given that our model implements active inference within a variational recurrent neural network, it is naturally suited for capturing temporal and dynamic aspects of action and language. A future extension of this work toward multirobot interaction under the framework of “collective active inference” or “federated active inference” may thus provide novel insights into the evolution of embodied language, moving beyond static object labeling toward dynamic and action-oriented communication.

## MATERIALS AND METHODS

In this section, we present the model architecture used in this study. The current model, as well as our earlier work (*34*), extends a study by Kawahara *et al.* (*33*). That study demonstrated that curiosity-driven reinforcement learning can be achieved within the framework of active inference (*27*, *56*), in which motor behavior is reinforced in the direction that minimizes expected free energy. More details are shown in the “Free energy principle, active inference, and Kawahara model” section of the Supplementary Materials, along with a brief introduction of the free energy principle and active inference.

### Used model

The current model, as well as our previous one (*34*), extends the approach proposed by Kawahara *et al.* (*33*) by implementing both the forward model and actor-critic using a variational recurrent neural network (*57*) to deal with temporal complexity and stochasticity inherent in robot-environment interactions.

The expected free energy *G* can be computed as

$$\begin{array}{c} G_{t}=-\underset{\text{Curiosity}}{\underbrace{\eta D_{\mathrm{KL}}\left[q\left(z_{t}|o_{t},a_{t-1},h_{t-1}\right)\|p\left(z_{t}|a_{t-1},h_{t-1}\right)\right]}}\\ -\underset{\text{Extrinsic reward}}{\underbrace{r\left(s_{t},a_{t}\right)}}-\underset{\text{Entropy}}{\underbrace{aH\left[\pi_{\phi }\left(a_{t}|h_{t}\right)\right]}} \end{array}$$ (3)

This equation is derived by replacing *w*, the probabilistic model learning parameter used in eq. S9, with *z*, the probabilistic model state. The weighting coefficients η and α are introduced to scale the contributions of the curiosity and motor entropy terms, respectively. The complexity term is computed as KLD between the approximated posterior distribution and the prior distribution over the latent variables at each time step. Both distributions are modeled as Gaussian distribution with time-dependent means and standard deviations. The approximated posterior is conditioned on the current sensory observation and the previous latent control variable, while the prior is conditioned only on the previous latent control variable. The resulting KLD thus reflects the information gain from that sensory observation, which is driven by the motor command executed at the previous time step. Therefore, exploration of more novel situations (i.e., curiosity-driven exploration) tends to result in higher information gain through larger complexity. The motor entropy in the third term of Eq. 3 reflects the expected uncertainty of the policy and is computed as the negative expected log probability of generating a motor command $a_{t}$ conditioned on the latent control variable $h_{t}$.

By adopting an analogous approach to the Kawahara model, the policy for generating a motor command $a_{t}$ is trained to minimize the expected free energy $G_{t}$ (Eq. 3) through reinforcement learning using the Soft Actor-Critic algorithm (*45*). Accordingly, the $Q_{t}$ value is updated as

$$\begin{array}{c} Q_{t}=r_{t}+\eta D_{\mathrm{KL}}\left[q\left(z_{t}|o_{t},h_{t-1}\right)\|p\left(z_{t}|h_{t-1}\right)\right]+aH\left[\pi_{\phi }\left(a_{t+1}|h_{t}\right)\right]\\ +\gamma \left(1-{\text{done}}_{t}\right)E_{o_{t+1}∼D,a_{t+1}∼\pi_{\phi }}\left[Q_{\bar{\theta }}\left(o_{t+1},a_{t+1}\right)\right] \end{array}$$ (4)

The first term $r_{t}$ represents the extrinsic reward. The second term $D_{\mathrm{KL}}\left[q\left(z_{t}|o_{t},h_{t-1}\right)\|p\left(z_{t}|h_{t-1}\right)\right]$ is the intrinsic reward for curiosity, scaled by a positive coefficient η. The third term $H\left[\pi_{\phi }\left(a_{t+1}|h_{t}\right)\right]$ is the intrinsic reward for motor entropy, scaled by a positive coefficient α. The fourth term is the bootstrapped estimate of the next step’s value, $\hat{Q_{t+1}}$, which is weighted by a discount rate parameter γ∈[0,1]. The variable ${\text{done}}_{t}$ is zero for all steps except the episode’s final step, where it is set to one. This restrains the definition of $Q_{t}$ to steps within the episode.

The critic $Q_{\theta }\left(o_{t+1},a_{t+1}\right)$ is trained to generate $\hat{Q_{t}}$, approximation of $Q_{t}$. The target critic $Q_{\bar{\theta }}\left(o_{t+1},a_{t+1}\right)$ is maintained for stability in the critic’s training. Initially identical to the critic, the target critic is updated via Polyak averaging such that $\bar{\theta }\leftarrow \tau \theta +\left(1-\tau \right)\bar{\theta }$ with τ∈[0,1]. The actor $\pi_{\phi }\left(o_{t}\right)$ is trained to generate motor commands $a_{t}$, which maximize the critic’s predictions of value. To mitigate positive bias, it is common to train multiple separate critics (each with its own target critic) (*45*). The actor is trained using the minimum predicted value across critics. Our model uses two separate critics.

The forward model is trained dynamically over the course of exploratory learning by optimizing the model parameters ψ to minimize the evidence free energy $F_{\psi }$ (eq. S3) after each trial episode. The exact implementation of this process is described in the “Details of the model architecture” in the Supplementary Materials.

#### Robot actions

The robot and the objects were simulated in PyBullet, the Python physics simulator. Each wheel’s velocity was bounded within the range of [−10, 10] m/s. For scale, the robot’s body is a cube measuring 2 m along each dimension (length, width, and height). The robot’s arm features two joints: yaw, which rotates left or right within a range of [−30°, 30°], and pitch, which rotates forward or upward within a range of [0°, 90°]. For smooth movement, the robot’s wheel and arm velocities were implemented with linear interpolation from the current to the target velocities.

We defined success criteria for each action category, which determined whether or not the robot earned an extrinsic reward by completing a goal. The distance between the robot and an object was measured from the object’s center to the center of the robot’s body. The robot was considered to be “facing the object” when the angular deviation between the robot’s forward direction and the line connecting it to the object was less than 15°.

Watch: The robot faces the object between 6 and 10 m of distance. This must be maintained for six steps in a row. Be near: The robot faces the object with a distance of less than 6 m without touching the object. This must be maintained for five steps in a row. Touch the top: The robot’s hand contacts with the object, while the center of the hand is at least 3.75 m above the floor. This must be maintained for three steps in a row. Push forward: The robot pushes the object farther than 0.1 m with respect to the robot’s facing direction. This must be maintained for three steps in a row. Push left: The robot pushes the object to the robot’s left farther than 0.2 m, while the robot’s wheels have velocities below 5 m/s (requiring the use of the arm). This must be maintained for three steps in a row. Push right: Same as Push left but in the opposite direction. There are constraints in rewarding for actions, which are described in the “Constraints in performing actions” section in the Supplementary Materials.

## Supplementary Materials

### The PDF file includes:

Tables S1 to S8 Supplementary Text Legend for movie S1 Figs. S1 to S7 References
