# Supplementary material for: Curiosity-driven development of action and language in robots through self-exploration
Source: Sci Adv. 2026 Jul 23;12(30):eaee7533. doi: 10.1126/sciadv.aee7533 (PMC13394400; doi:10.1126/sciadv.aee7533)
Supplement: Supplementary file 1 — Tables S1 to S8 Supplementary Text Legend for movie S1 Figs. S1 to S7 References [file sciadv.aee7533_sm.pdf]

Science Advances
AAAS

Supplementary Materials for

Curiosity-driven development of action and language in robots through self-exploration

Theodore J. Tinker *et al.*

Corresponding author: Jun Tani, jun.tani@oist.jp

Sci. Adv. **12**, eaee7533 (2026)
DOI: 10.1126/sciadv.aee7533

The PDF file includes:

Tables S1 to S8
Supplementary Text
Legend for movie S1
Figs. S1 to S7
References

Other Supplementary Material for this manuscript includes the following:

Movie S1

Variable	Definition
o_t	Observation at time t
$o_{t,i}$	i^{th} part of observation o_t
$o_{t,v}$	Our agent's $o_{t,0}$, vision
$o_{t,ta}$	$o_{t,1}$, touch
$o_{t,p}$	$o_{t,2}$, proprioception
$o_{t,cw}$	$o_{t,3}$, command voice
$o_{t,fw}$	$o_{t,4}$, feedback voice
a_t	Motor Command
r_t	Extrinsic reward
$done_t$	Final step of episode
$mask_t$	Steps inside episode
R	Recurrent replay buffer
π	Actor
ϕ	Actor's parameters
Q	Critic
θ	Critic's parameter
$\bar{\theta}$	Target critic's parameter
τ	Critic's soft update coefficient

Variable	Definition
f	Forward model
ψ	Forward model parameters
γ	Discount for future rewards
α	Importance of motor entropy
η	Importance of curiosity
η_i	η for i^{th} part of observation
$p(z_t)$, $q(z_t)$	Prior, approximated posterior
μ, σ	Mean, standard deviation
h_t	RNN hidden state
z_t	Sample from posterior
enc_i	Encoder for $o_{t,i}$
ψ_i^{enc}	f parameters for enc_i
dec_i	Decoder for $o_{t,i}$
ψ_i^{dec}	f parameters for dec_i
MLP_i^{prior}	Multilayer for prior for $o_{t,i}$
MLP_i^{post}	Multilayer for approximated posterior for $o_{t,i}$

Table S1. Definitions of variables.

Supplementary Text

For future reference, table S1 includes definitions for relevant variables.

Movie S1. Training example. Compares a robot with *all curiosity* mid-training and after training.

Free Energy Principle, Active Inference, and Kawahara Model

We begin by describing predictive coding and active inference (AIF), which are grounded in the free energy principle (FEP) (*58*). The FEP posits that biological and artificial agents maintain

their existence by minimizing variational free energy, which is an upper bound on sensory surprise. In perception, this process is often instantiated as predictive coding (*58, 59, 60, 61*), wherein internal models reconstruct sensory inputs by updating beliefs or latent variables by minimizing the reconstruction errors. More formally, this is minimizing evidence free energy defined for past observations. In motor command generation, the FEP framework extends to AIF (*25, 27*), where agents minimize the future prediction error (quantified as expected free energy) by optimizing the latent variables and motor commands in the future. These two processes are tightly coupled and must be considered jointly in embodied cognition systems.

We next introduce the work of Kawahara et al. (*33*), who proposed a novel reinforcement learning (RL) scheme that integrates (AIF).

In the Bayesian framework, the true posterior probability distribution $p(z_t|o_t)$ over latent variables z_t, conditioned on sensory observations o_t, is given by Bayes' rule:

$$p(z_t|o_t) = \frac{p(o_t|z_t)p(z_t)}{\int p(o_t, z_t)dz}$$

Here, $p(z_t)$ denotes the prior. The denominator, called the evidence, is usually intractable; to overcome this, variational Bayes introduces an approximation of the posterior $q(z_t)$. This is optimized to minimize the Kullback-Leibler divergence (KLD) between the approximated posterior $q(z_t)$ and the true posterior $p(z_t|o_t)$.

$$\begin{aligned} D_{KL}[q(z_t)||p(z_t|o_t)] &= \int q(z_t) \log \frac{q(z_t)}{p(z_t|o_t)} dz_t \\ &= \int q(z_t) \log \frac{q(z_t)p(o_t)}{p(z_t, o_t)} dz_t \\ &= \int q(z_t) \log \frac{q(z_t)p(o_t)}{p(z_t)p(o_t|z_t)} dz_t \qquad \text{(S1)} \\ &= F + \log p(o_t) \qquad \text{(S2)} \end{aligned}$$

The term F here is the evidence free energy, equal to

$$F_t = \underbrace{D_{KL}[q(z_t)||p(z_t)]}_{\text{Complexity}} - \underbrace{\mathbb{E}_{q(z_t)}[\log p(o_{t+1}|z_t)]}_{\text{Accuracy}} . \qquad \text{(S3)}$$

Since $p(o_t)$ is constant for a given sensory observation, minimizing KLD is equivalent to minimizing F_t. Therefore, the optimal posterior approximation is:

$$q^*(z_t) = \underset{q(z_t)}{\arg\min}\, F_t \tag{S4}$$

In active inference, the agent minimizes expected free energy G_τ at a future time step $\tau \geq t+1$. This is the expected value of the evidence free energy under the predictive distribution of future outcomes (*33*).

$$\begin{aligned}
G_\tau &= \mathbb{E}_{p(o_\tau|z_\tau)}[F] \\
&= \mathbb{E}_{p(o_\tau|z_\tau)}[\int q(z_\tau)\log\frac{q(z_\tau)}{p(o_\tau, z_\tau)}dz] \\
&= \mathbb{E}_{p(o_\tau|z_\tau)}[\mathbb{E}_{q(z_\tau)}[\log\frac{q(z_\tau)}{p(z_\tau|o_\tau)} - \log p(o_\tau)]].
\end{aligned} \tag{S5}$$

Recalling that $q(z_\tau|o_\tau)q(o_\tau) = q(o_\tau, z_\tau)$, we approximate:

$$\begin{aligned}
G_\tau &\approx \mathbb{E}_{q(o_\tau,z_\tau)}[\log\frac{q(z_\tau)}{q(z_\tau|o_\tau)} - \log p(o_\tau)] \\
&= -\mathbb{E}_{q(o_\tau,z_\tau)}[\log\frac{q(z_\tau|o_\tau)}{q(z_\tau)}] - \mathbb{E}_{q(o_\tau)}[\log p(o_\tau)] \\
&= -\underbrace{\mathbb{E}_{q(o_\tau)}\overbrace{[D_{KL}[q(z_\tau|o_\tau)||q(z_\tau)]]}^{\text{Bayesian Surprise}}}_{\text{Epistemic Value or Mutual Information}} - \underbrace{\mathbb{E}_{q(o_\tau)}[\log p(o_\tau)]}_{\text{Extrinsic Value}}.
\end{aligned} \tag{S6}$$

The first term, $I(z_\tau, o_\tau) = \mathbb{E}_{q(o_\tau)}[D_{KL}[q(z_\tau|o_\tau)||q(z_\tau)]]$, is the mutual information (or Bayesian surprise). This depicts expected information gain based on new sensory observation o_τ, and can be expressed as:

$$I(z_\tau, o_\tau) = \underbrace{H(z_\tau)}_{\text{Shannon Entropy}} - \underbrace{H(z_\tau|o_\tau)}_{\text{Conditional Entropy}}.$$

The second term, $p(o_\tau)$, represents log-likelihood of the preferred sensory observation. This is specified as the extrinsic reward designed by the experimenters. For the intrinsic value to reflect mutual information or information gain, and the extrinsic value to reflect expected free energy, is the same as the way shown by Friston's group in the study of active inference (*27, 56*). Separating o_t into o_t and a_t, we rewrite the expected free energy as:

$$
\begin{aligned}
G_\tau &= -\mathbb{E}_{q(o_\tau,a_\tau,z_\tau)}[\log \frac{p(z_\tau|o_\tau,a_\tau)}{q(z_\tau)}] - \mathbb{E}_{q(o_\tau,a_\tau)}[\log p(o_\tau,a_\tau)] \\
&= -\mathbb{E}_{q(o_\tau,a_\tau,z_\tau)}[\log \frac{p(z_\tau,a_\tau|o_\tau)}{q(z_\tau)p(a_\tau|o_\tau)}] - \mathbb{E}_{q(o_\tau,a_\tau)}[\log p(o_\tau,a_\tau)] \\
&\approx -\mathbb{E}_{q(o_\tau,a_\tau,z_\tau)}[\log \frac{q(z_\tau|o_\tau)q(a_\tau|o_\tau,z_\tau)}{q(z_\tau)p(a_\tau|o_\tau)}] - \mathbb{E}_{q(o_\tau,a_\tau)}[\log p(o_\tau,a_\tau)] \\
&= -\mathbb{E}_{q(a_\tau|o_\tau,z_\tau)q(o_\tau)}[D_{KL}[q(z_\tau|o_\tau)||q(z_\tau)]] \\
&- \mathbb{E}_{q(o_\tau,z_\tau)}[D_{KL}[q(a_\tau|o_\tau,z_\tau)||p(a_\tau|o_\tau)]] \\
&- \mathbb{E}_{q(o_\tau,a_\tau)}[\log p(o_\tau,a_\tau)]. \qquad \text{(S7)}
\end{aligned}
$$

Kawahara et al. (*33*) developed a forward model $f_w(o_\tau, a_\tau) \rightarrow \widehat{o}_{\tau+1}$ which learns to predict the future sensory observation $o_{\tau+1}$ based on o_τ and a_τ using a Bayesian Neural Network (BNN) (*62*). In this type of model, the network parameters w_τ are treated as random variables defined with Gaussian distribution. These parameters serve as latent causes of observed sensory transitions and can be interpreted as random latent variables for the generative model. Therefore, w_τ corresponds to z_τ.

Let the approximate posterior be defined as $q_\psi = \mathcal{N}(w_\tau|\mu,\sigma)$, with parameters $\psi = \{\mu,\sigma\}$. In this setting, the actor π_ϕ of a SAC can be trained to approximate $\pi_\phi(a_\tau|o_\tau) \approx q(a_\tau|o_\tau, w_\tau)$. This allows rewriting the expected free energy as:

$$
\begin{aligned}
G(o_\tau,a_\tau) = &-\mathbb{E}_{q(a_\tau|o_\tau,w_\tau)q(o_\tau)}[D_{KL}[q(w_\tau|o_\tau)||q(w_\tau)]] \\
&- \mathbb{E}_{q(o_\tau,a_\tau)}[D_{KL}[\pi_\phi(a_\tau|o_\tau)||p(a_\tau|o_\tau)]] \\
&- \mathbb{E}_{q(o_\tau,a_\tau)}[\log p(o_\tau,a_\tau)]. \qquad \text{(S8)}
\end{aligned}
$$

Let us interpret the prior preference $\log p(o_\tau, a_\tau)$ as the extrinsic reward $r(s_\tau, a_\tau)$, where s_τ is the true environmental state. Bring focus to the current time step by setting $\tau = t$. Because the forward model trains to predict o_{t+1}, we can further rewrite the expected free energy as:

$$
\begin{aligned}
G(o_t, a_t) &= -D_{KL}[q_\psi(w_t|o_{t+1})||q_\psi(w_t)] - \log p(o_t, a_t) \\
&- D_{KL}[\pi_\phi(a_t|o_t)||p(a_t|o_t)] \\
&= -D_{KL}[q_\psi(w_t|o_{t+1})||q_\psi(w_t)] - \log p(o_t, a_t) \\
&- \int \pi_\phi(a_t|o_t) \log \pi_\phi(a_t|o_t) da_t + \int \pi_\phi(a_t|o_t) \log p(a_t|o_t) da_t \\
&= -\underbrace{D_{KL}[q_\psi(w_t|o_{t+1})||q_\psi(w_t)]}_{\text{Curiosity}} - \underbrace{r(s_t, a_t)}_{\text{Extrinsic Reward}} - \underbrace{\mathcal{H}(\pi_\phi(a_t|o_t))}_{\text{Entropy}} - \underbrace{\mathbb{E}_{\pi_\phi(a_t|o_t)}[\log p(a_t^*|o_t)]}_{\text{Imitation}}
\end{aligned}
\tag{S9}
$$

Because w_τ represents the robot's probabilistic knowledge of its environment, the first term of Eq. S9 can be said to represent the robot's gain in knowledge based on information acquired in a new sensory observation.

In summary, the forward model is trained to minimize the evidence free energy F (Eq. S3) by accurately reconstructing sensory observations and minimizing posterior complexity based on past experiences. Meanwhile, the actor-critic pair is trained to minimize expected free energy G, which includes an inverted complexity term (i.e., curiosity) and motor entropy to encourage exploration. This leads to emergent tension in an adversarial relationship: the actor is encouraged to maximize information gain by increasing the KL divergence between prior and posterior, while the forward model trains to minimize that same term. This establishes a dynamic push-pull effect, driving self-organized exploration. Please note that the imitation term in Eq. S9 depends on external demonstrations or expert policies; this term is ignored in our study, which focuses on self-exploration.

From this formulation of expected free energy, the Q-value can be updated as:

$$
\begin{aligned}
Q(t) &= r_t + \eta D_{KL}[q_\psi(w_t|o_{t+1})||q_\psi(w_t)] + \\
&\gamma(1 - done_t)\mathbb{E}_{o_{t+1}\sim D, a_{t+1}\sim\pi_\phi}[Q_{\bar{\theta}}(o_{t+1}, a_{t+1})] + \alpha\mathcal{H}(\pi_\phi(a_{t+1}|o_{t+1}))
\end{aligned}
\tag{S10}
$$

Here, $\eta > 0$ and $\alpha > 0$ are hyperparameters weighting the intrinsic reward based on the curiosity and the motor entropy, respectively.

In our experiments, each episode ended after 30 steps, or terminated earlier if the agent successfully executed the command. Completed episodes are stored in a recurrent replay buffer, which can hold up to 256 episodes. When the buffer is full, the buffer discards the oldest episodes to accommodate new episodes. To ensure uniform episode length, all episodes were padded to 30 steps with empty transitions. Hence, transitions are stored with the form $\{o_t, a_t, r_t, o_{t+1}, done_t, mask_t\}$, where $mask_t = 1$ for real transitions, and $mask_t = 0$ for empty transitions added for padding. After each episode, a batch of 32 episodes was sampled from the buffer and used to train the forward model, actor, and critics. During training, loss terms were multiplied by $mask_t$, removing the influence of empty transitions.

Details of the Model Architecture

This subsection explains further details about the model architecture employed in this current study. As noted earlier, the present architecture extends our previous model (*34*), which is described in the "Free energy principle, Active Inference, and Kawahara Model" section of the Supplementary Materials. The primary extension involves the use of separate random latent variables, encoders, and decoders for each sensory modality. This design allows the model to process multiple types of sensation independently, including vision, tactile input, proprioception, command voice, and feedback voice. Regarding proprioception, our model uses an encoder for the 4-dimensional motor command, which includes motor velocities for the robot's two wheels and two joint angles in its arm. The full architecture of the proposed model is shown in Fig. S1.

Computation in this architecture proceeds as follows:

1. The 4-dimensional motor command from the previous time step is fed into the motor command encoder, producing an encoded motor command vector.

2. The prior distribution for each observational modality in the current time step is computed using the encoded motor command vector and the previous latent control variable. Distinctly, the prior distribution for the command voice, which is constant and not impacted by the robot's motor command, is assigned a normal Gaussian distribution.

3. The sensory observation for each modality is fed through its corresponding encoder, computing its modality-specific encoded vector.

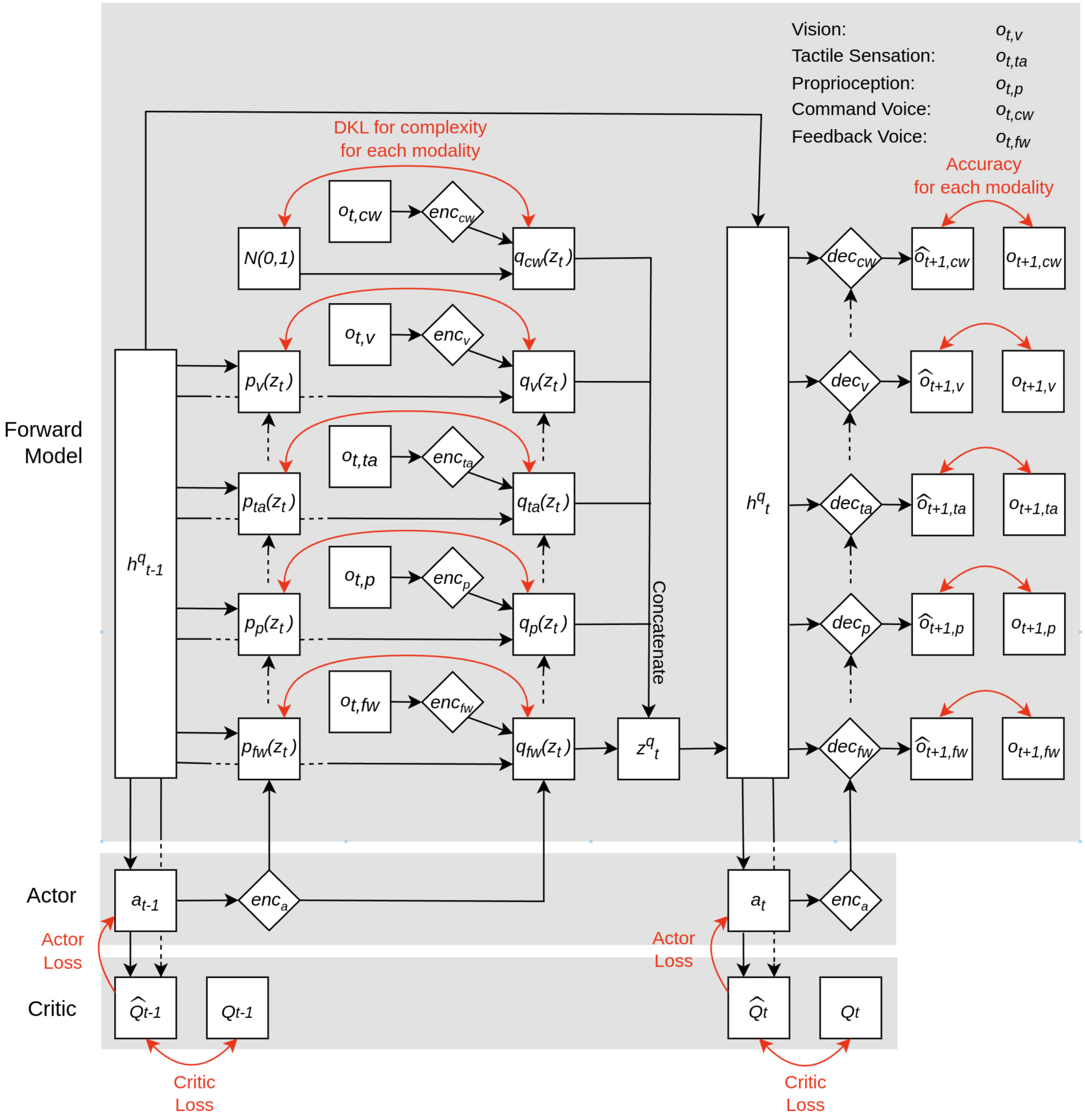

Fig. S1. **The details of the proposed model architecture.**

4. The approximated posterior distribution for each modality is approximated using its sensory encoded vector, encoded motor command vector, and the previous latent control variable. Distinctly, the posterior distribution for the constant command voice is approximated based only on the encoding of the command voice alone, separating it from the motor command and latent control variable.

5. All posterior vectors from the current time step are concatenated across all modalities, then

sampled and combined with the previous latent control variable to compute the current latent control variable.

6. The motor command for the current time step is generated from the current latent control variable using the actor (policy network).

7. The model predicts the next sensory observation for each modality using the current latent control variable and the current motor command, passed through the corresponding sensory decoders. Although the command voice is constant, it is still predicted; this ensures that the command voice is conserved in the latent control variable for the benefit of the actor and critic portions of the model.

8. The Q_t value is updated according to Eq. 4.

9. If the episode terminates at this step, the episode's data is saved in a recurrent replay buffer. A batch of information is sampled from the buffer to train the forward model, actor, and critic.

More thorough pseudocode is provided in the following section, utilizing variables found in table S1. Details of the encoders and decoders of each sensory modality (e.g., vision, tactile sensation, etcetera), as well as the motor command encoder, are described in the "Implementation details" section of the Supplementary Materials.

Pseudocode

The algorithm depicted in algorithm pages 1 through 3 is pseudocode representing the usage of the proposed model.

Implementation details

Vision

The robot visually senses the environment in the direction the robot faces with a $16 \times 16 \times 4$ image, with the four channels being red, green, blue, and distance. See Fig. S2.

In our proposed model, in order to make the approximated posterior for visual sensations, images are flattened and encoded using a linear neural network with Parametric Rectified Linear

Algorithm 1 Pseudocode for Proposed Model (Part One)

Initialize forward model f_ψ, actor π_ϕ, critic Q_θ, replay buffer R ▷ Multiple critics may be used
Initialize target critic weights $\bar{\theta} \leftarrow \theta$ ▷ One target critic for each critic
for epoch = 0, M **do**
▷ In each epoch, the agent plays one episode and trains with a batch of episodes
  Initialize hidden state $h^q_{-1} = 0.0$ and motor command $a_{-1} = 0.0$ ▷ Begin new episode
  Receive observation with n parts, $o_0 = o_{0,0}, ..., o_{0,n}$
  for t = 0, T **do** ▷ Steps in episode
    for i = 0, n **do** ▷ Parts in observation
      $o^{enc}_{t,i} \leftarrow \text{enc}_i(o_{t,i})$ ▷ Encode observation part
      $\mu^q_{t,i}, \sigma^q_{t,i} \leftarrow \text{MLP}^{post}_i(h^q_{t-1}||a_{t-1}||o^{enc}_{t,i})$
      ▷ Posterior inner state distribution; || denotes concatenation
      ▷ The posterior of the constant command voice does not utilize h^q_{t-1} or a_{t-1}
      $z^q_{t,i} \sim q(z_{t,i}) = \mathcal{N}(\mu^q_{t,i}, \sigma^q_{t,i}))$ ▷ Sample posterior inner state
    end for
    $h^q_t \leftarrow \text{RNN}(h^q_{t-1}, z^q_{t,0}||...||z^q_{t,n})$ ▷ Advance h^q
    Execute motor command $a_t \leftarrow \pi_\phi(h^q_t)$ to receive o_{t+1}, r_t, and $done_t$
    ▷ If $done_t$, stop episode
  end for
  Store episode's transitions $(o_{0:T+1}, a_{-1:T}, r_{0:T}, done_{0:T})$ in R ▷ Save episode

Note: Algorithm continues.

Unit activation (PReLU). To generate a prediction of the next image, h^q_t and a^{enc}_t are concatenated and decoded with another linear neural network, shaped into a $16x16x4$ tensor, and finished with a convolutional layer. See details in table S2.

Touch

The second part of the sensory observation is the tactile sensation of touch. This is represented by one value between 0 and 1 for each of the robot's 16 sensors. Each value is equal to the fraction of time in the previous step during which the respective sensor was in contact with an object. See Fig. S3.

Algorithm 1 Pseudocode for Proposed Model (Part 2)

Sample batch of episodes $(o_{0:T+1}, a_{-1:T}, r_{0:T}, done_{0:T}, mask_{0:T})$ from R ▷ Sample batch

Initialize forward model hidden state $h^q_{-1} = 0.0$ ▷ Begin training

for t = 0, T+1 **do** ▷ Steps in advancing forward model with batch

  Initialize $P_{t-1} = 0.0$ ▷ Begin tracking curiosity

  for i = 0, n **do** ▷ Parts in observation

    $\mu^p_{t,i}, \sigma^p_{t,i} \leftarrow \text{MLP}^{prior}_i(h^q_{t-1} || a_{t-1})$ ▷ Prior inner state distribution

    ▷ Prior of the constant command voice is assigned a normal Gaussian distribution

    $o^{enc}_{t,i} \leftarrow \text{enc}_i(o_{t,i})$ ▷ Encode observation part

    $\mu^q_{t,i}, \sigma^q_{t,i} \leftarrow \text{MLP}^{post}_i(h^q_{t-1} || a_{t-1} || o^{enc}_{t,i})$ ▷ Posterior inner state distribution

    ▷ The posterior of the constant command voice does not utilize h^q_{t-1} and a_{t-1}

    $P_{t-1} += \eta_i D_{KL}[q(z_{t,i}) || p(z_{t,i})]$

    ▷ Compare these prior and posterior to add to curiosity

    $z^q_{t,i} \sim q(z_{t,i}) = \mathcal{N}(\mu^q_{t,i}, \sigma^q_{t,i}))$ ▷ Sample posterior inner state

  end for

  $h^q_t \leftarrow \text{RNN}(h^q_{t-1}, z^q_{t,0} || ... || z^q_{t,n})$ ▷ Advance h^q

end for

for t = 0, T+1 **do** ▷ Steps in advancing critics with batch

  $\widehat{Q}_t \leftarrow Q_\theta(h^q_{t-1}, a_t)$ ▷ Predict Q value

  $a'_t \leftarrow \pi_\phi(h^q_t)$ ▷ Make new motor command with actor

  if $t > 1$ **then** ▷ After first step, make target Q-values with target critics

    $\overline{Q_t} \leftarrow Q_{\bar{\theta}}(h^q_{t-1}, a'_t)$ ▷ Get target critic's Q value

    $Q_t \leftarrow r_t + P_t + \gamma(1 - done_t)(\overline{Q_t} - \alpha\mathcal{H}(\pi_\phi(a'_t|o_t)))$

    ▷ Make target Q value (eq. S10)

  end if

end for

Note: Algorithm continues.

In our proposed model, in order to make the approximated posterior for tactile sensation, the tensor is encoded using a linear neural network with PReLU. To generate a prediction of the next tactile sensation, h^q_t and a^{enc}_t are concatenated and decoded with another linear neural network.

Algorithm 1 Pseudocode for Proposed Model (Part 3)

Initialize $F = 0.0$ ▷ Initiate free energy

for i = 0, n **do** ▷ Parts in observation

$F += (\beta_i D_{KL}[q(z_{0:T,i})||p(z_{0:T,i})] - \upsilon_i \mathbb{E}_{q(z_{0:T,i})}[\log p(o_{1:T+1,i}|z_{0:T,i})]) * mask_{0:T}$

▷ Add complexity, subtract accuracy (see eq. S3)

end for

$\psi \leftarrow \psi - \lambda_\psi \frac{\partial F}{\partial \psi}$ ▷ Train forward model

$J_Q(\theta) \leftarrow (\widehat{Q}_{0:T} - Q_{1:T+1})^2 * mask_{0:T}$ ▷ Critic loss

$\theta \leftarrow \theta - \lambda_Q \hat{\nabla}_\theta J_Q(\theta)$ ▷ Train critics

$\bar{\theta} \leftarrow \tau\theta + (1-\tau)\bar{\theta}$ ▷ Update target critics

$J_\pi(\phi) \leftarrow (-Q_\theta(o_{0:T}, a'_{0:T}) - \alpha\mathcal{H}(\pi_\phi(a'_{0:T}|o_{0:T}))) * mask_{0:T}$

▷ Actor loss. Use the lowest Q-value among critics.

$\phi \leftarrow \phi - \lambda_\pi \hat{\nabla}_\phi J_\pi(\phi)$ ▷ Train actor

if utilizing dynamic α with target entropy $\bar{\mathcal{H}}$ **then**

$J_\alpha(\alpha) \leftarrow \log(\alpha) \cdot (\mathcal{H}(\pi(a_{0:T}|o_{0:T})) - \bar{\mathcal{H}}) * mask_{0:T}$ ▷ Alpha loss

$\log(\alpha) \leftarrow \log(\alpha) - \lambda_\alpha \hat{\nabla}_\alpha J_\alpha(\alpha)$ ▷ Train alpha

end if

end for

Note: Algorithm is completed.

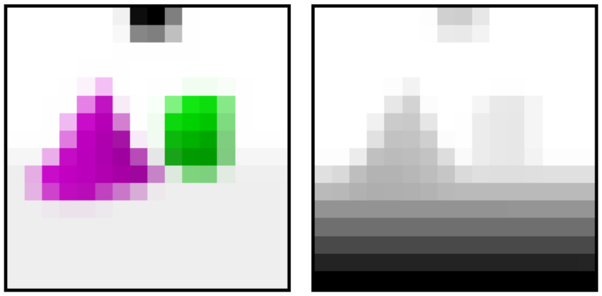

Fig. S2. **The agent's vision, $o_{t,v}$.** The robot is facing a magenta cone and a green pillar. The robot also sees part of its hand. The image on the left depicts the red, green, and blue channels. The image on the right depicts the distance.

Layer	Type	Activation	Details
		**Encoder,** enc_v	
1	Flatten		Shape (16, 16, 4) to shape (1024).
2	Linear	PReLU	To shape (128).
		**Decoder**, dec_v	
1	Linear	BatchNorm2d, PReLU	From shape (264) to shape (8 * 8 * 64).
2	Reshaping		To shape (8, 8, 64).
3	CNN	Tanh	Kernel size 3, reflective padding 1.<br>To shape (8, 8, 8).
4	Pixel Shuffle		To shape (16, 16, 4).

Table S2. **Encoder and decoder of agent's visual sensations,** $o_{t,v}$**.**

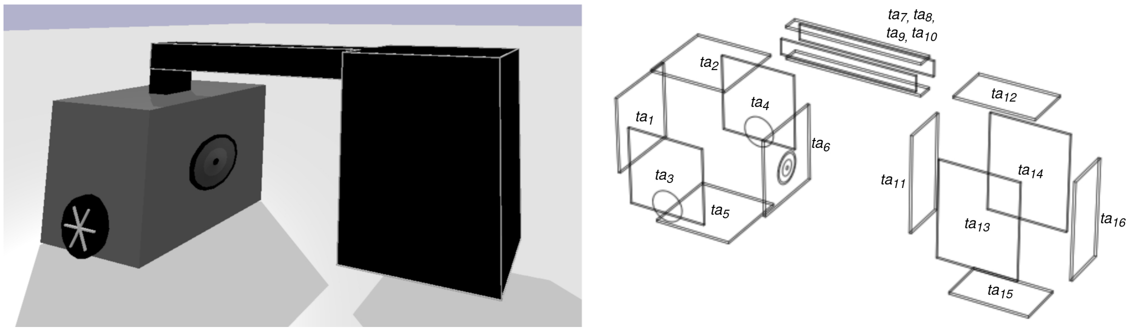

Fig. S3. **The agent's sensors for tactile sensations of touch,** $o_{t,ta}$**.** The robot has 16 sensors, which are planes on the surface of the robot's body, arm, and hand. The camera and wheels are marked just for clarity.

See details in table S3.

Proprioception

The third part of the sensation is the angle and velocity of the arm's joints. (The velocity of the joint may not match the robot's motor commands, because collisions with objects may restrain it.) This consists of a tensor with four values between 0 and 1: two joint angles and two joint velocities. Each value is the normalized proportion of the respective variable between its minimum and maximum range.

Layer	Type	Activation	Details
**Encoder,** enc_{ta}			
1	Linear	BatchNorm2d, PReLU	From shape (16) to shape (20).
**Decoder,** dec_{ta}			
1	Linear	BatchNorm2d, TanH	From shape (264) to shape (16).<br>Result added to 1 and divided by 2<br>for values between 0 and 1.

Table S3. Encoder and decoder of agent's tactile sensations, $o_{t,ta}$**.**

In our proposed model, in order to make the approximated posterior for sensation of proprioception, the tensor is encoded using a linear neural network with PReLU. To generate a prediction of the next proprioception, h_t^q and a_t^{enc} are concatenated and decoded with another linear neural network. See details in table S4.

Layer	Type	Activation	Details
**Encoder,** enc_{po}			
1	Linear	BatchNorm2d, PReLU	From shape (4) to shape (4).
**Decoder,** dec_{po}			
1	Linear	BatchNorm2d, TanH	From shape (264) to shape (4).<br>Result added to 1 and divided by 2<br>for values between 0 and 1.

Table S4. Encoder and decoder of agent's sensation of proprioception, $o_{t,p}$**.**

Voices

The fourth and fifth parts of the sensation are the command voice and the tutor-feedback voice, which were described briefly in the Results section. Both voices are sequences of one-hot vectors. Table S5 displays the 18 words (including silence) and their indexes in the one-hot vectors. For example, the command "Watch the Red Pillar" is represented by

English Word Indexes	
**Index**	**Word**
0	(Silence)
1	Watch
2	Be Near
3	Touch the Top
4	Push Forward
5	Push Left
6	Push Right
7	Red
8	Green
9	Blue
10	Cyan
11	Magenta
12	Yellow
13	Pillar
14	Pole
15	Dumbbell
16	Cone
17	Hourglass

Table S5. **English words and indexes**. The English words used and their positions in one-hot vectors.

$$\begin{gathered}[0,1,0,0,0,0,0,0,0,0,0,0,0,0,0,0,0,0] \\ [0,0,0,0,0,0,0,1,0,0,0,0,0,0,0,0,0,0] \\ [0,0,0,0,0,0,0,0,0,0,0,0,0,1,0,0,0,0]. \end{gathered} \tag{S11}$$

If the robot has not performed any action, then the feedback voice is only one one-hot vector indicating silence:

$$[1,0,0,0,0,0,0,0,0,0,0,0,0,0,0,0,0,0]. \tag{S12}$$

In our proposed model, in order to make the approximated posteriors for the sensations of each voice, the tensors are encoded using separate embeddings, recurrent neural networks, and linear layers. Note that these RNNs are "nested" within the forward model's RNN, such that each of the robot's steps includes three steps of interpreting each voice. See Fig. S4. To generate a prediction of the next voices, h_t^q and a_t^{enc} are concatenated and decoded using separate recurrent neural networks for the command voice and feedback voice. See details in table S6.

Layer	Type	Activation	Details
**Encoders,** enc_{cw} and enc_{fw}			
1	Embedding	PReLU	From shape (Sequence-length, 18) to shape (Sequence-length, 8).
2	Linear	PReLU	To shape (Sequence-length, 64).
3	GRU	PReLU	To shape (64).
4	Linear	PReLU	To shape (256).
**Decoders,** dec_{cw} and dec_{fw}			
1	Linear	BatchNorm2d, PReLU	From shape (264) to shape (192).
2	Reshaping		To shape (3, 64).
3	GRU	PReLU	To shape (3, 64).
4	Linear		To shape (3, 18).

Table S6. **Encoder and decoder of agent's voice sensation,** $o_{t,cw}$ **and** $o_{t,fw}$**.**

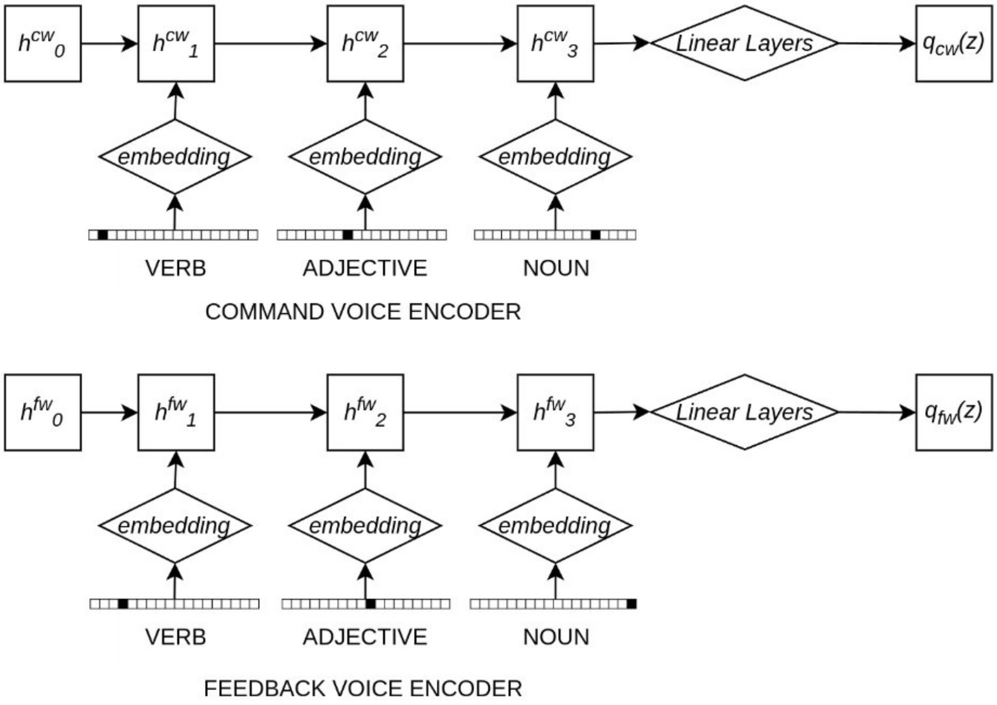

Fig. S4. **Recurrent steps of command voice and feedback voice.**

Motor Command Encoder

For usage in the forward model, the robot's motor commands a_t are encoded into a_t^{enc} with a linear neural network with PReLU. See details in table S7.

Layer	Type	Activation	Details
**Encoder,** enc_a			
1	Linear	PReLU	From shape (4) to shape (8).

Table S7. **Encoding motor command for forward model.**

Constraints in Performing Actions

In each step, the robot can only perform one of the six actions. This is implemented using definitions of actions and action prioritization. The actions "watch," "be near," and "touch the top" cannot be performed simultaneously because of requirements regarding distance from the object and touching the object. The actions "push left" and "push right" cannot be performed simultaneously because of the directions of movements. If the robot satisfies the requirements for "touch the top," we reject the actions "push forward," "push left," or "push right." If the robot is performing "push forward" and "push left" or "push right," we accept only the action with the greatest distance pushed.

Details of Experiments

Experiment 1

In Experiment 1 we trained robots with three levels of curiosity: *no curiosity*, *sensory-motor curiosity*, and *all curiosity*. Table S8 reports the values of the hyperparameter η for each of the four components of the sensory observations subject to exploration. These parameters represent the relative contribution of each sensory component to the robot's intrinsic curiosity.

Fig. S5 shows rolling success-rates for robots in the case of *no curiosity* which were trained for 120,000 epochs. Although these robots attained 80.8 percent success, it took twice the training duration of robots in the case of *all curiosity* in Fig. 3.

Name	η_{vision}	η_{touch}	$\eta_{proprioception}$	$\eta_{feedback}$
No Curiosity	0	0	0	0
Sensory-Motor Curiosity	.03	1	1	0
All Curiosity	.03	1	1	.3

Table S8. **Hyperparameters for three types of agents.**

Fig. S6 and Fig. S7 show rolling success-rates for robots utilizing a conventional baseline model architecture employing the Soft Actor-Critic (SAC) algorithm (*45*) with both the actor and critics implemented with GRU (*46*). This baseline model showed its best performance when the number of learning parameters was set as 2,874,013. (Our proposed architecture has 3,998,493 learning parameters.) We applied the same hyperparameters for entropy used by our proposed models. When trained on all six action categories, the baseline model performed poorly, with a success-rate of nearly 0%; see Fig. S6. However, in Fig. S7, the baseline model demonstrated competency with an overall success-rate of roughly 59% with unlearned goals when trained only on the tasks "watch," "be near," and "push forward." However, that success-rate is still worse than our proposed model utilizing curiosity-driven exploration when trained with all six tasks.

Statistical Analysis of U-Shaped Patterns

To quantify U-shaped learning in exception-handling, we scored the U-shaped structure of success-rate trajectories identifying non-monotonic developmental patterns consistent with representational redescription (*11*). The method combines robust smoothing, normalized scaling, and piecewise isotonic regression to fit a two-phase model with a central valley.

Consider one robot's rolling-average success rate over training epochs for goals which are exceptions. The U-shape score is computed as follows:

1. **Burn-in removal.** The first 10% of training data is removed to avoid initialization noise.

2. **Smoothing.** The curve is smoothed using a Savitzky–Golay filter with a window length of approximately 3% of the series, reducing spurious local fluctuations.

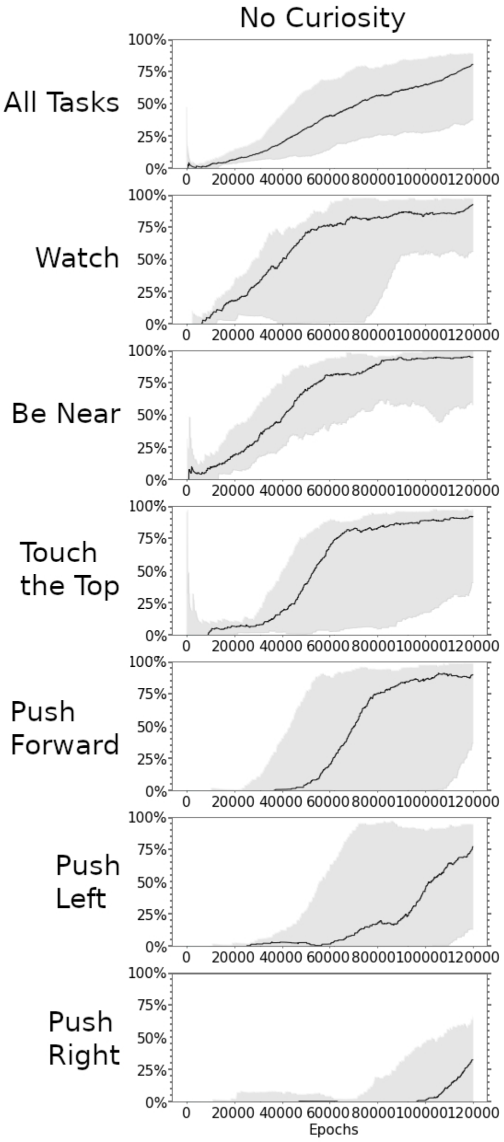

Fig. S5. **Rolling success-rates over extended training in the case of *no curiosity.*** Success-rates of robots with *no curiosity* when trained for 120,000 epochs, which is twice the duration of robots in Fig. 3.

3. **Normalization.** The smoothed curve is linearly scaled to the $[0, 1]$ range using the 5th and 95th percentiles to ensure robustness across success-rate ranges.

4. **Valley localization.** The minimum point i_M is located between 20% and 80% of the sequence length.

5. **Piecewise isotonic regression.** For each candidate split point k near the valley (within ±25%

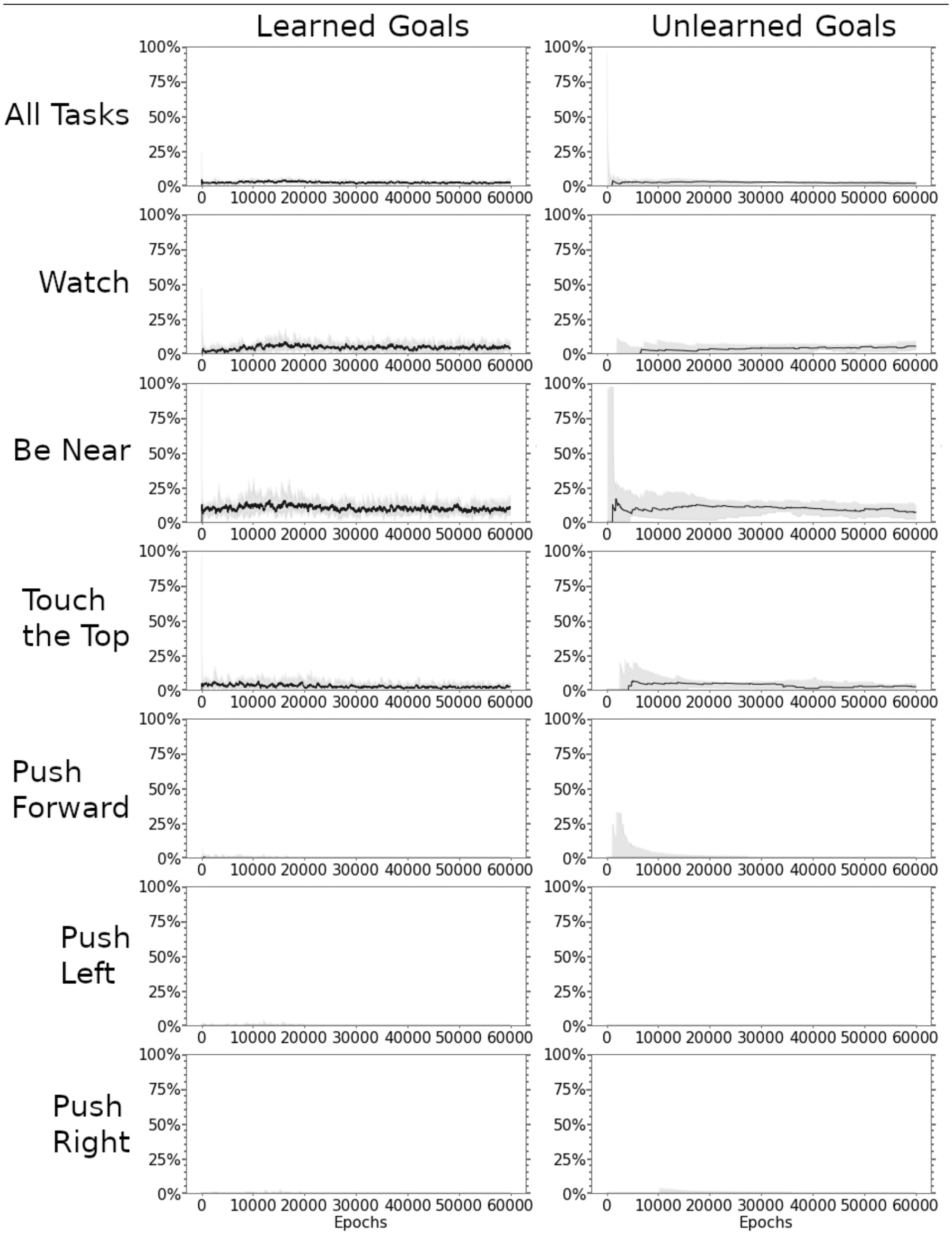

Fig. S6. **Rolling success-rates for robots using the baseline recursive SAC model using all six tasks.** Poor success-rates of robots using a more traditional architecture.

of the series), the left segment is fit with a decreasing isotonic regression and the right segment with an increasing isotonic regression. A cost function is minimized:

$$\text{Cost}(k) = \text{MSE}(k) + \lambda \cdot (\text{drift from valley})^2 \cdot \text{MSE}_{\text{base}},$$

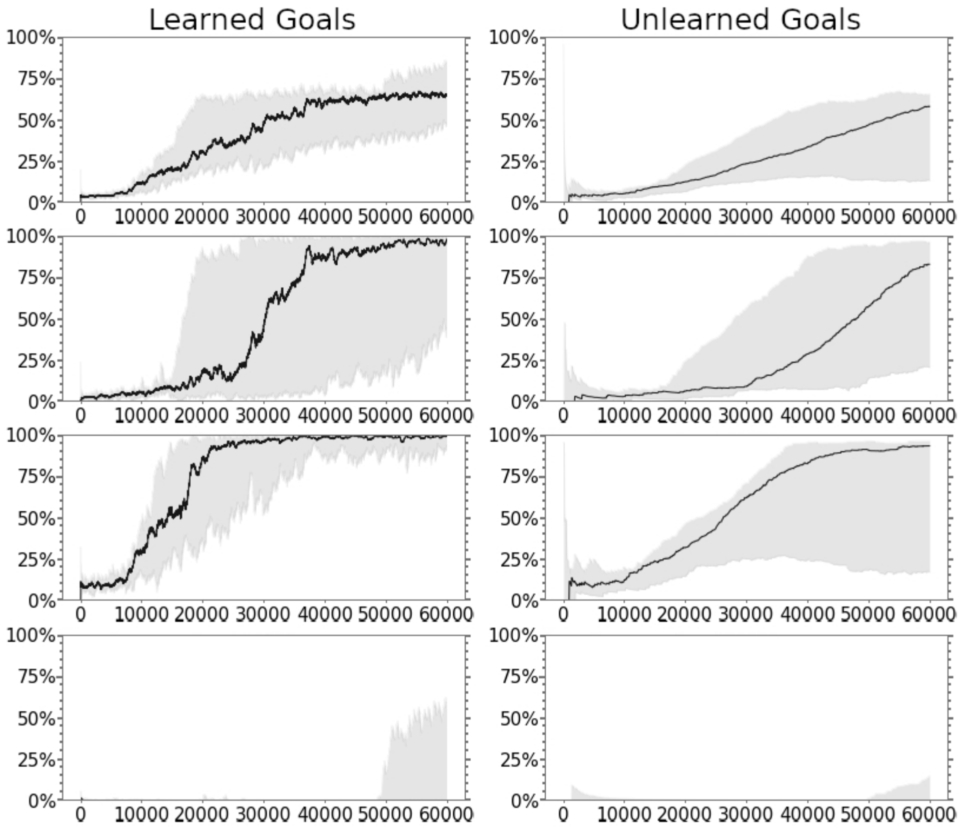

Fig. S7. **Rolling success-rates for robots using the baseline recursive SAC model using just three tasks.** Success-rates of robots using a more traditional architecture.

where $\lambda = 2.0$ penalizes drifting too far from the identified valley. Indices of k define the left peak i_L and right peak i_R.

6. **Score calculation.** If the best split passes depth and width criteria (minimum 3% depth, 6% width), a composite U-score is computed:

$$\text{U-score} = 0.6 \cdot \text{improvement} + 0.25 \cdot \text{depth} + 0.15 \cdot \text{width},$$

where:

- *Improvement* is the fractional MSE reduction relative to the best monotonic baseline fit.
- *Depth* is the drop from the valley to the 90th percentile of the surrounding peaks.
- *Width* is the relative proportion of the sequence before/after the valley.

7. **Index reporting.** Indices of the left peak i_L, valley i_M, and right peak i_R are marked with red vertical lines in Figure 7.

To compare robots trained with exceptions and without exceptions, we computed U-shape scores for each robot individually and compared the two groups using a one-tailed Welch's t-test (unequal variances):

$$t = \frac{\bar{x}_1 - \bar{x}_2}{\sqrt{\frac{s_1^2}{n_1} + \frac{s_2^2}{n_2}}}.$$

The resulting test statistic confirmed that robots trained with exceptions exhibited significantly stronger U-shaped profiles than those without, with $p = 0.0001$.

REFERENCES

1. M. Tomasello, *Constructing A Language: A Usage-Based Theory of Language Acquisition* (Harvard Univ. Press, Cambridge, MA, 2003).

2. L. R. Gleitman, The structural sources of verb meanings. *Lang. Acquis.* **1**, 3–55 (1990).

3. P. Bloom, *How Children Learn the Meanings of Words* (MIT Press, 2000).

4. L. B. Smith, E. Thelen, Development of word learning: An embodied perspective. *Dev. Rev.* **25**, 205–244 (2005).

5. G. Frege, "On sense and reference" in *Translations from the Philosophical Writings of Gottlob Frege*, M. Black, P. Geach, Eds. (Blackwell, 1952), pp. 56–78, original work published 1892.

6. R. Montague, "Universal grammar" in *Formal Philosophy: Selected Papers of Richard Montague*, R. H. Thomason, Ed. (Yale Univ. Press, 1974), pp. 222–246, original manuscript 1970.

7. Z. G. Szabó, "Compositionality" in *The Stanford Encyclopedia of Philosophy*, E. N. Zalta, Ed. (Metaphysics Research Lab, Stanford Univ., 2017), fall 2017 edition.

8. J. A. Fodor, Z. W. Pylyshyn, Connectionism and cognitive architecture: A critical analysis. *Cognition* **28**, 3–71 (1988).

9. B. M. Lake, M. Baroni, "Generalization without systematicity: On the compositional skills of sequence-to-sequence recurrent networks," in *Proceedings of the 35th International Conference on Machine Learning (ICML)*, vol. 80 of *Proceedings of Machine Learning Research* (PMLR, 2018), pp. 2873–2882.

10. N. Chomsky, *Rules and Representations* (Columbia Univ. Press, 1980).

11. A. Karmiloff-Smith, *Beyond Modularity: A Developmental Perspective on Cognitive Science* (MIT Press, Cambridge, MA, 1992).

12. D. E. Rumelhart, J. L. McClelland, "On learning the past tenses of English verbs" in *Parallel Distributed Processing, Volume 2: Explorations in the Microstructure of Cognition*, J. L. McClelland, D. E. Rumelhart, Eds. (MIT Press, 1986), pp. 216–271.

13. K. Plunkett, V. Marchman, U-shaped learning and frequency effects in a multilayer perceptron: Implications for child language acquisition. *Cognition* **38**, 43–102 (1991).

14. V. Marchman, K. Plunkett, From U-shaped learning to systematicity: A connectionist account of English past tense acquisition. *Cognition* **48**, 279–304 (1993).

15. J. L. Elman, E. Bates, M. H. Johnson, A. Karmiloff-Smith, D. Parisi, K. Plunkett, *Rethinking Innateness: A Connectionist Perspective on Development* (MIT Press, 1996).

16. D. Mareschal, T. R. Shultz, Computational developmental psychology. *Trends Cogn. Sci.* **5**, 178–185 (2001).

17. M. Asada, K. F. MacDorman, H. Ishiguro, Y. Kuniyoshi, Cognitive developmental robotics as a new paradigm for the design of humanoid robots. *Rob. Auton. Syst.* **37**, 185–193 (2001).

18. Y. Kuniyoshi, S. Sangawa, Early motor development from partially ordered neural-body dynamics: Experiments with a cortico-spinal-musculo-skeletal model. *Biol. Cybern.* **95**, 589–605 (2006).

19. G. Sandini, G. Metta, J. Konczak, Developmental robotics: Insights from developmental psychology on robotic learning. *Prog. Brain Res.* **164**, 327–346 (2007).

20. T. J. Prescott, P. F. Dominey, Synthesizing the temporal self: Robotic models of episodic and autobiographical memory. *Philos. Trans. R Soc. Lond. B Boil. Sci.* **379**, 20230415 (2024).

21. A. Cangelosi, T. Riga, Simulation of language and action learning in a multi-agent environment. *Proc. IEEE* **92**, 396–401 (2004).

22. Y. Sugita, J. Tani, Cross-situational learning of words and sentences: A developmental robotics experiment. *Proc. IEEE* **92**, 428–442 (2005).

23. A. Taniguchi, T. Taniguchi, T. Inamura, Spatial concept acquisition for a mobile robot that integrates self-localization and unsupervised word discovery from spoken sentences. *IEEE Trans. Cogn. Dev. Syst.* **8**, 285–297 (2016).

24. R. Vijayaraghavan, D. Roy, A. Cangelosi, Grounding language learning in embodied interaction: A review of approaches and challenges. *Front. Robot. AI* **8**, 625891 (2021).

25. K. Friston, J. Mattout, J. Kilner, Action understanding and active inference. *Biol. Cybern.* **104**, 137–160 (2011).

26. G. Pezzulo, F. Rigoli, K. J. Friston, Hierarchical active inference: A theory of motivated control. *Trends Cogn. Sci.* **22**, 294–306 (2018).

27. T. Parr, K. J. Friston, Generalised free energy and active inference. *Biol. Cybern.* **113**, 495–513 (2019).

28. C. J. C. H. Watkins, P. Dayan, Q-learning. *Mach. Learn.* **8**, 279–292 (1992).

29. R. S. Sutton, A. G. Barto, *Reinforcement Learning: An Introduction* (MIT Press, ed. 2, 2018).

30. Z. Fountas, N. Sajid, P. Mediano, K. Friston, Deep active inference agents using Monte-Carlo methods. *Adv. Neural Inf. Proces. Syst.* **33**, 11662–11675 (2020).

31. B. Millidge, Deep active inference as variational policy gradients. *J. Math. Psychol.* **96**, 102348 (2020).

32. K. Ueltzhöffer, Deep active inference. *Biol. Cybern.* **112**, 547–573 (2018).

33. D. Kawahara, S. Ozeki, I. Mizuuchi, "A curiosity algorithm for robots based on the free energy principle," in *2022 IEEE/SICE International Symposium on System Integration (SII)* (Narvik, Norway, 2022).

34. T. J. Tinker, K. Doya, J. Tani, Intrinsic rewards for exploration without harm from observational noise: A simulation study based on the free energy principle. *Neural Comput.* **36**, 1854–1885 (2024).

35. P.-Y. Oudeyer, F. Kaplan, V. V. Hafner, Intrinsic motivation systems for autonomous mental development. *IEEE Trans. Evol. Comput.* **11**, 265–286 (2007).

36. J. Schmidhuber, "A possibility for implementing curiosity and boredom in model-building neural controllers," in *Proceedings of the International Conference on Simulation of Adaptive Behavior: From Animals to Animats* (IEEE, 1991), pp. 222–227.

37. P. K. Kuhl, Early language acquisition: Cracking the speech code. *Nat. Rev. Neurosci.* **5**, 831–843 (2004).

38. K. E. Adolph, J. M. Franchak, Learning to move, moving to learn: A quarter century of progress in studying infant motor development. *Child Dev. Perspect.* **9**, 214–219 (2015).

39. B. A. Goldfield, J. S. Reznick, Early lexical acquisition: Rate, content, and the vocabulary spurt. *J. Child Lang.* **17**, 171–183 (1990).

40. M. Carpenter, K. Nagell, M. Tomasello, *Social Cognition, Joint Attention, and Communicative Competence from 9 to 15 Months of Age* (Monographs of the Society for Research in Child Development, 1998).

41. J. Bruner, *Child's Talk: Learning to Use Language* (Oxford Univ. Press, 1983).

42. L. S. Vygotsky, *Mind in Society: The Development of Higher Psychological Processes* (Harvard Univ. Press, 1978).

43. S. Pinker, *The Language Instinct* (William Morrow, 1994).

44. E. S. Spelke, K. D. Kinzler, Core knowledge. *Dev. Sci.* **10**, 89–96 (2007).

45. T. Haarnoja, A. Zhou, P. Abbeel, S. Levine, "Soft actor-critic: Off-policy maximum entropy deep reinforcement learning with a stochastic actor," in *Proceedings of the 35th International Conference on Machine Learning*, vol. 80 of *Proceedings of Machine Learning Research*, J. Dy, A. Krause, Eds. (PMLR, 2018), pp. 1861–1870, https://proceedings.mlr.press/v80/haarnoja18b.html.

46. Z. Yang, H. Nguyen, “Recurrent off-policy baselines for memory-based continuous control,” in *Deep Reinforcement Learning Workshop, NeurIPS 2021* (NeurIPS, 2021).

47. L. Gerken, S. Knight, Infants generalize from just (the right) four words. *Cognition* **143**, 187–192 (2015).

48. L. Gerken, C. Dawson, R. Chatila, J. Tenenbaum, Surprise! Infants consider possible bases of generalization for a single input example. *Dev. Sci.* **18**, 80–89 (2015).

49. A. Cangelosi, M. Schlesinger, *Developmental Robotics: From Babies to Robots* (MIT Press, 2015).

50. O. Lipschits, R. Geva, An integrative model of parent–infant communication development. *Child Dev. Perspect.* **18**, 137–144 (2024).

51. C. Suarez-Rivera, C. S. Tamis-LeMonda, Pathways from social contingency to infant language learning. *Philos. Trans. R. Soc. B Biol. Sci.* **381**, 20240359 (2026).

52. E. V. Clark, *First Language Acquisition* (Cambridge Univ. Press, 2009).

53. L. Steels, “A self-organizing spatial vocabulary” in *Artificial Life IV* (MIT Press, 1995), pp. 179–184.

54. S. Li, R. Miikkulainen, “Evolving artificial language using evolutionary reinforcement learning,” in *Proceedings of the 8th International Conference on the Simulation of Adaptive Behavior* (MIT Press, 2006), pp. 182–191.

55. T. Taniguchi, T. Nagai, T. Nakamura, Symbol emergence in cognitive developmental systems: A survey. *IEEE Trans. Cogn. Dev. Syst.* **11**, 494–516 (2019).

56. K. Friston, T. FitzGerald, F. Rigoli, P. Schwartenbeck, J. O. Doherty, G. Pezzulo, Active inference and learning. *Neurosci. Biobehav. Rev.* **68**, 862–879 (2016).

57. J. Chung, K. Kastner, L. Dinh, K. Goel, A. Courville, Y. Bengio, A recurrent latent variable model for sequential data. *Adv. Neural Inf. Proces. Syst.* **28**, 2980–2988 (2015).

58. K. J. Friston, A theory of cortical responses. *Philos. Trans. R. Soc. Lond. B Biol. Sci.* **360**, 815–836 (2005).

59. R. P. Rao, D. H. Ballard, Predictive coding in the visual cortex: A functional interpretation of some extra-classical receptive-field effects. *Nat. Neurosci.* **2**, 79–87 (1999).

60. J. Hohwy, *The Predictive Mind* (OUP Oxford, 2013).

61. A. Clark, *Surfing Uncertainty: Prediction, Action, and The Embodied Mind* (Oxford Univ. Press, 2015).

62. C. Blundell, J. Cornebise, K. Kavukcuoglu, D. Wierstra, "Weight uncertainty in neural network," in *International Conference on Machine Learning* (PMLR, 2015), pp. 1613–1622.
